# Identification of developmental stage and anatomical fraction contributions to cell wall recalcitrance in switchgrass

**DOI:** 10.1186/s13068-017-0870-5

**Published:** 2017-07-15

**Authors:** Jacob D. Crowe, Nicholas Feringa, Sivakumar Pattathil, Brian Merritt, Cliff Foster, Dayna Dines, Rebecca G. Ong, David B. Hodge

**Affiliations:** 1 Department of Chemical Engineering and Materials Science, Michigan State University, East Lansing, MI USA; 2 Complex Carbohydrate Research Center, University of Georgia, Athens, GA USA; 3 Bioenergy Science Center, Oak Ridge National Laboratory, Oak Ridge, TN USA; 4 DOE-Great Lakes Bioenergy Research Center, Michigan State University, East Lansing, MI USA; 5 Department of Chemical Engineering, Michigan Technological University, Houghton, MI USA; 6 Department of Biosystems & Agricultural Engineering, Michigan State University, East Lansing, MI USA; 7 Department of Civil, Environmental and Natural Resources Engineering, Luleå University of Technology, Luleå, Sweden

**Keywords:** Switchgrass, Recalcitrance, Cell wall glycans, Alkaline pretreatment

## Abstract

**Background:**

Heterogeneity within herbaceous biomass can present important challenges for processing feedstocks to cellulosic biofuels. Alterations to cell wall composition and organization during plant growth represent major contributions to heterogeneity within a single species or cultivar. To address this challenge, the focus of this study was to characterize the relationship between composition and properties of the plant cell wall and cell wall response to deconstruction by NaOH pretreatment and enzymatic hydrolysis for anatomical fractions (stem internodes, leaf sheaths, and leaf blades) within switchgrass at various tissue maturities as assessed by differing internode.

**Results:**

Substantial differences in both cell wall composition and response to deconstruction were observed as a function of anatomical fraction and tissue maturity. Notably, lignin content increased with tissue maturity concurrently with decreasing ferulate content across all three anatomical fractions. Stem internodes exhibited the highest lignin content as well as the lowest hydrolysis yields, which were inversely correlated to lignin content. Confocal microscopy was used to demonstrate that removal of cell wall aromatics (i.e., lignins and hydroxycinnamates) by NaOH pretreatment was non-uniform across diverse cell types. Non-cellulosic polysaccharides were linked to differences in cell wall response to deconstruction in lower lignin fractions. Specifically, leaf sheath and leaf blade were found to have higher contents of substituted glucuronoarabinoxylans and pectic polysaccharides. Glycome profiling demonstrated that xylan and pectic polysaccharide extractability varied with stem internode maturity, with more mature internodes requiring harsher chemical extractions to remove comparable glycan abundances relative to less mature internodes. While enzymatic hydrolysis was performed on extractives-free biomass, extractible sugars (i.e., starch and sucrose) comprised a significant portion of total dry weight particularly in stem internodes, and may provide an opportunity for recovery during processing.

**Conclusions:**

Cell wall structural differences within a single plant can play a significant role in feedstock properties and have the potential to be exploited for improving biomass processability during a biorefining process. The results from this work demonstrate that cell wall lignin content, while generally exhibiting a negative correlation with enzymatic hydrolysis yields, is not the sole contributor to cell wall recalcitrance across diverse anatomical fractions within switchgrass.

**Electronic supplementary material:**

The online version of this article (doi:10.1186/s13068-017-0870-5) contains supplementary material, which is available to authorized users.

## Background

Development of renewable sources of fuels and chemicals is necessary to improve energy security and mitigate climate change effects resulting from greenhouse gas emissions [1, 2]. Lignocellulosic biomass, which includes dedicated bioenergy crops and agricultural residues, is a widely available and largely untapped source of reduced carbon in the production of renewable fuels and chemicals [3]. A current major challenge in deconstructing plant cell walls are plant-evolved mechanisms for resisting microbial and animal degradation of plant cell walls, termed biomass recalcitrance [4]. Within a feedstock, abundance and density of cell type, structure and substitution of cell wall polymers, and organization of polymers within the cell wall can all contribute to biomass recalcitrance [4].

Plant cell walls account for the majority of lignocellulosic feedstock dry mass, and are comprised of primary and in some cases secondary cell walls depending upon the tissue type. Primary cell walls are found in growing cells, and contain cellulose microfibrils, pectins, and hemicellulose polysaccharides. Secondary cell walls are significantly thicker than primary cell walls, provide mechanical strength and structural reinforcement to the cells, are found predominantly in fiber and vascular cells post cell enlargement [5], and represent the majority of the mass of lignocellulosic feedstocks. Secondary cell walls contain cellulose microfibrils, hemicelluloses, and lignin, an aromatic heteropolymer comprising phenylpropanoid subunits. In monocot grasses, the primary hemicellulose components are glucuronoarabinoxylans (“xylans”) containing arabinosyl, acetyl, and uronosyl substitutions on a β-1,4 linked xylan backbone [6]. The arabinofuranosyl residues in grass xylans can also be covalently linked via ferulic acid ester crosslinks to lignin or other xylans, and this phenomenon has been hypothesized to play an important role in promoting cell wall rigidity and decelerating expansion [7]. Mixed-linkage glucan and xyloglucans are also present as minor hemicellulose components in cell walls, and may account for a minor fraction of non-cellulosic glucan found in mature tissues [6, 8]. Lignin content and composition are often cited as one of the predominant contributors of cell wall recalcitrance [9]; however, there also exists a body of literature that has examined the role of hemicelluloses [10], structural pectins [11], and polymer interactions within the cell wall as other significant contributors to cell wall recalcitrance [12].

Switchgrass (*Panicum virgatum* L.) is a well-studied C_4_ perennial grass envisioned as a promising herbaceous feedstock to supply North American cellulosic biorefineries along with other monocot grasses such as miscanthus (*Miscanthus* × *giganteus*), and corn stover (*Zea mays*) [13, 14]. Switchgrass is an attractive feedstock due to high production yields, low energy and nutrient inputs, wide adaptability, and short growing time [15]. Switchgrass grows as a clonal modular organism, with the tiller forming the main clonal module and phytomers consisting of multiple shoot meristems. Shoots comprise leaf blades, sheath leaves, stem nodes and internodes, auxiliary buds, and ligules [16]. Grasses such as switchgrass grow through elongation of stem internodes, each of which is linked at the base node to a single leaf sheath and blade. Analysis of individual internodes, as opposed to bulk plant material, is useful as internodes represent sequential stages of maturity during plant development, affecting cell wall composition and abundance of cell type within biomass [17]. Basal internodes tend to have greater lignin and cellulose content coupled with lower digestibility compared to internodes near the top of a single tiller [18]. Tissue maturity in relation to harvest times may impact switchgrass digestibility [19], including time left on field post maturation [20]. In addition, there are significant differences in compositions of stem, sheath, and leaf anatomical fractions, with the stem generally containing more lignin and being less digestible compared to the leaf [21, 22]. The distribution, structure, and extractability of non-cellulosic cell wall glycans in miscanthus stem and leaf organs sampled at distinct maturities indicated that hemicellulose structure and extractability varied with tissue maturity, and may account for significant non-lignin contributions to recalcitrance [23].

Within-plant heterogeneity for graminaceous feedstocks such as switchgrass has important impacts on several important processing variables for a biorefining process. Ranges of particle size distribution, composition, hygroscopicity and drainability, chemical pretreatment effectiveness, and extent of enzymatic hydrolysis can all result from differing responses of biomass to comminution. Physical fractionation, employed either on-field during harvest or at the biorefinery, is one potential route to addressing feedstock challenges to potentially improve agronomic, logistics, and processing–conversion outcomes. Limited work has been published on biomass fractionation in the context of pretreatment and hydrolysis. In one study, disc refining followed by air classification of corn stover yielded pith and depithed stover fractions that were subjected to dilute acid pretreatment or mild base pretreatment, quantifying mass losses and sugar release response during acid treatment [24]. Other work identified significant differences in enzymatic hydrolysis response of corn stover stems compared to other fractions [25–27].

Studies investigating tissue-specific responses have been assessed for liquid hot water pretreatment of corn stover [28] and wheat straw [29], although the only industrially relevant fractionation approach that have been studied are alteration of leaf-stem ratio in wheat straw [30]. With AFEX pretreatment, the impact of knife-milling and sieving of corn stover [31] as well manual separation of stem, sheath, leaf, and cob have been assessed [32], and found to yield fractions with differing compositions and responses to deconstruction.

The objective of this study is to characterize within-plant heterogeneity by identifying key compositional differences between switchgrass anatomical fractions at different internode maturities and relating compositional differences to enzymatic digestibility response following mild alkaline pretreatment. Structural differences are evaluated by localization of phenolics within tissue cross sections before and after alkaline pretreatment using confocal microscopy, while hemicellulose and pectin structure and extractability are evaluated using glycome profiling following chemical extraction.

## Methods

### Field conditions and biomass preparation

**Fig. 1:**
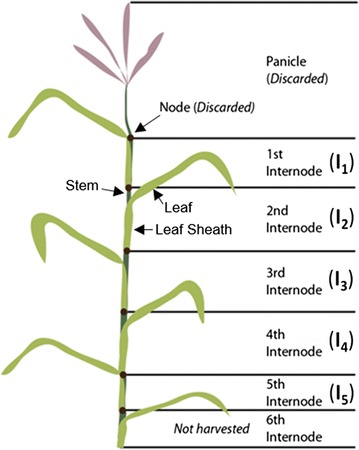
Schematic of switchgrass tillers used for this study. Tillers were harvested above either the 5th or 6th internode, and then separated into internodes, discarding the nodes and panicle. Internodes were further manually subdivided into leaves, leaf sheaths, and stems. Diagram is representative and not to scale

Switchgrass (var. “Forestburg,” upland ecotype) was manually harvested post-anthesis above either the 5th or 6th internode (Fig. 1) from a field at Michigan State University (East Lansing, MI; 42°42′48.80′′N by 84°28′1.41′′W) between 17 and 19 September, 2014. During harvest, intact harvested tillers were dried indoors at room temperature. After harvest was completed, all tillers were dried overnight in a 45 °C oven. Internodes were then manually separated into leaves, leaf sheaths, and stems, while the nodes and panicle were discarded (Fig. 1). Whole fractions were air dried to a moisture content of ~5% (g H_2_O/g total), and particle size was reduced using a Wiley Mini-Mill (Thomas Scientific, Swedesboro, NJ, USA) to pass a 30-mesh screen. Samples were stored in dry, airtight bags until further use.

### Pretreatment conditions

Alkaline pretreatment was performed in 8 mL borosilicate glass tubes (Thermo Fisher Scientific, Waltham, MA, USA) with rubber lined caps. Alkali loading was performed at 10% (g NaOH/g dry biomass) loading at 10% (g biomass/g solution) loading, giving a 0.25 M NaOH concentration. Samples were vortexed and immersed in an 80 °C water bath for 1 h, with mixing every 15 min. After the elapsed pretreatment time, solids were separated from the alkaline liquor and washed with de-ionized water until pH neutral. Following washing, samples were rinsed with absolute ethanol and allowed to air dry at 45 °C to a moisture content <5%, with mass loss following pretreatment determined gravimetrically.

### Composition analysis

Extractives-free cell wall material (i.e., alcohol insoluble residue or “AIR”) was isolated following extraction and de-starching according to Foster et al. [33]. All composition procedures were performed on de-starched AIR, except for the determination of extractable sugars. Minor polysaccharide content (rhamnan, mannan, galactan, fucan, non-cellulosic glucan) was determined using the alditol acetate method with minor changes [33]. Cell wall acetate, glucan, xylan, arabinan, and Klason lignin content were determined for AIR using the NREL/TP 510-42618 protocol [34] with minor modifications [35]. Thioacidolysis was used to determine lignin monolignol yields [36]. Extractable free glucose, sucrose, and starch were determined on the original untreated biomass as described by Santoro et al. [37].

Alkaline saponification was used to determine the *p*-hydroxycinnamic acid content, as described previously [38], with modifications to the quantification method. Briefly, 0.5 g of biomass was treated with 25 mL of 3 M NaOH in a sealed pressure tube at 121 °C for 1 h to release esterified *p*-coumarate (*p*CA) and esterified or etherified ferulate (FA). Liquid samples were centrifuged at 13,000×*g* for 3 min. Next, 1 mL of alkaline liquor was taken, and pH adjusted to pH 1.5 using concentrated H_2_SO_4_ (Sigma-Aldrich Corp., St. Louis, MO, USA). Samples were centrifuged at 13,000×*g* for 3 min to remove any precipitated solids, and subsequently analyzed by HPLC (Agilent 1100 Series) with an Aminex HPX-87H column (Bio-Rad, Hercules, CA, USA) using a mobile phase of 5 mM H_2_SO_4_ with a 10% (v/v) ACN organic modifier. Quantification was performed using a diode-array detector with a UV wavelength of 210 nm. Standards containing FA and *p*CA ranged from 0.05 to 1 mg/mL. To account for precipitation of *p*CA and FA with pH adjustment, solubility with respect to pH curves were determined, with *R* ^2^ linear coefficients of 0.999 for both FA and *p*CA observed from the range of pH 1.4–1.8. Samples were analyzed in biological triplicate for reproducibility.

### Enzymatic hydrolysis

Enzymatic hydrolysis was performed at 2.5% solids loading (g AIR biomass/g solution) in 1.5 mL microcentrifuge tubes (Posi-Click, Denville Scientific, Holliston, MA). Enzymatic hydrolysis was performed using 10 mg protein/g glucan for pretreated biomass and 30 mg protein/g glucan for untreated, de-starched, extractives-free biomass using CTec3 and HTec3 (Novozymes A/S Bagsværd, Denmark), at a CTec3:HTec3 ratio of 2:1. A buffer solution of 50 mM citric acid (pH 5.20) was used to maintain pH, and samples were incubated at 50 °C with orbital mixing at 180 rpm for either 6 or 48 h. Samples were centrifuged at 13,000×*g* for 3 min post-incubation and filtered using 22 µm mixed cellulose-ester filters (EMD Millipore, Billerica, MA). Glucose and xylose were quantified by HPLC (Agilent 1100 Series) with an Aminex HPX-87H column (Bio-Rad, Hercules, CA, USA) with a mobile phase of 5 mM H_2_SO_4_. Glucose yield was determined based on quantified glucose released compared to total AIR cell wall glucan available (as glucose), including non-cellulosic glucan.

### Confocal microscopy

Air dried whole samples were sectioned by hand using a platinum tipped razor blade and prepared for imaging by washing with 50 mL of de-ionized water prior to immersing in water buffered at a pH of 7.0 by 20 mM Na_2_PO_4_ overnight. Pretreated samples were generated by NaOH pretreatment at 0.10 g NaOH/g biomass of whole cross sections, followed by washing with de-ionized water until pH neutral, and immersion in water buffered at a pH of 7.0 by 20 mM Na_2_PO_4_ overnight. Confocal fluorescent images were collected on an Olympus FluoView FV1000 Confocal Laser Scanning Microscope (Olympus America, Inc., Center Valley, PA, USA), configured on a fully automated Olympus IX81 inverted microscope. Images were recorded with a 10× UPlanFLN objective (NA 0.30). Blue and red autofluorescence signals were sequentially recorded, as shown in Additional file 1: Figure S1. Blue autofluorescence was excited using a 405 nm diode laser and emission was recorded using a 430–470 nm band pass emission filter. The red autofluorescence was excited using a 543 nm helium neon laser and emission was recorded using a 560 nm long pass emission filter. A series of confocal images (XYZ) were recorded through the thickness of each sample (approximately 50 µm). Images were collected in 10 μm intervals when using the 10× objective. Each confocal series was then compressed into a maximum intensity projection image and recorded in a TIF format. The color look-up table (LUT) for all images was modified with the blue display rescaled from 0–4095 to 350–800 and the red display rescaled from 0–4095 to 0–1200 to brighten images. All further processing was performed in ImageJ (http://imagej.nih.gov/ij/).

### Glycome profiling

Sequential cell wall extractions and glycome profiling of switchgrass anatomical fractions were carried out as described previously [39, 40] on AIR biomass. Plant glycan-directed mAbs were from laboratory stocks (CCRC, JIM, and MAC series) available at the Complex Carbohydrate Research Center [41]. A complete description of the mABs used in this study can be found in our prior work [42].

## Results

Switchgrass anatomical fractions sampled at different internodes throughout the plant representing differing levels of maturity were utilized in this work to develop an improved understanding of the impacts of within-plant heterogeneity in switchgrass on a potential cellulosic biofuels conversion process. Switchgrass samples were characterized with respect to composition and assessed for response to pretreatment and enzymatic hydrolysis with the goals of (1) identifying cell wall properties contributing to observed differences in cell wall recalcitrance, (2) understanding how the differences in composition and recalcitrance are distributed throughout the plant, and (3) potentially use this understanding to inform both plant breeding and harvesting strategies to optimally couple the plant feedstock to a conversion process.

### Anatomical fraction- and internode-specific recalcitrance

**Fig. 2:**
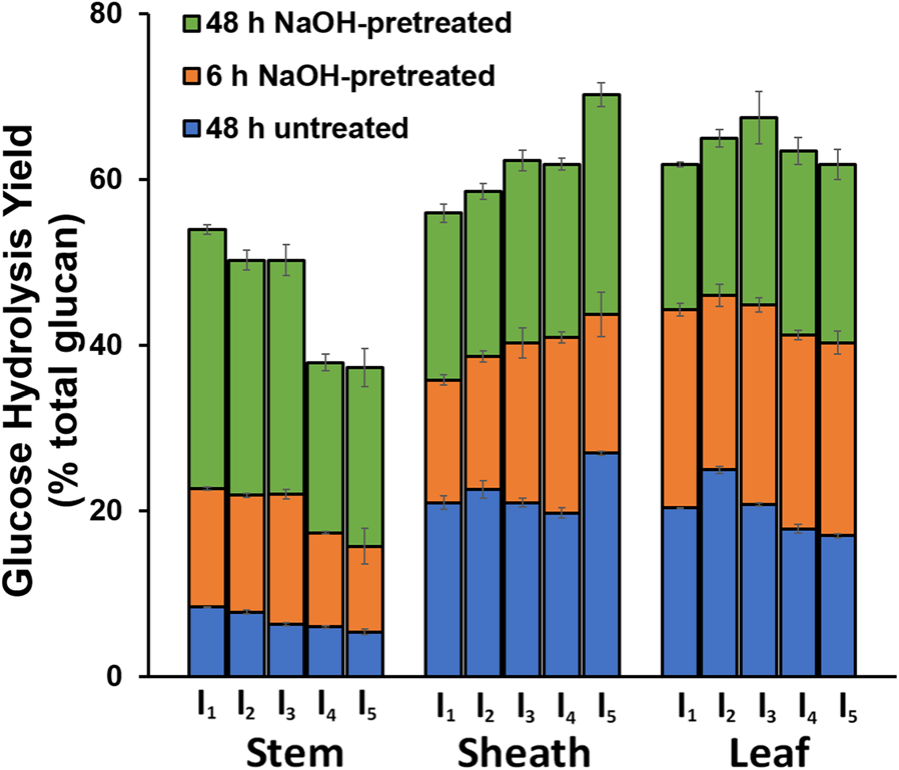
Enzymatic hydrolysis glucose yields of untreated and alkaline pretreatment of anatomical fractions. Enzymatic hydrolysis of untreated biomass was performed on AIR + de-starched biomass, while alkaline pretreatment was performed using 10% w/w NaOH loading at 80 °C for 1 h. Glucose hydrolysis yields are presented as the percent of total cell wall glucan released as monomeric glucose. Enzyme loading was 30 mg protein/g glucan for untreated fractions and 10 mg protein/g glucan for alkaline pretreated fractions with a ratio of 2:1 CTec3 to HTec3. Replicates (*n* = 3) are displayed as averages with standard deviations

Enzymatic hydrolysis before or after mild NaOH pretreatment was performed on extractives-free (AIR), de-starched cell wall material from three switchgrass anatomical fractions (leaves, leaf sheath, stem) as a function of internode (Fig. 2).

From the results in Fig. 2, lower glucose yields for both untreated and NaOH-pretreated cases of stem internodes indicate that stem fractions are significantly more recalcitrant compared to leaf sheath (referred to as sheath) and leaf internodes. Previous work using liquid hot water pretreatment of switchgrass also found that pretreated leaves were more digestible than stem internodes [43]. Also of note, the mass of above-ground biomass in switchgrass is known to be primarily leaves, with stems and leaf sheaths each comprising on the order of half the mass of the leaves [43]. Stem internodes also demonstrated a clear decrease in enzymatic hydrolysis yields with increasing tissue maturity (i.e., from I_1_ to I_5_) for both the untreated and NaOH-pretreated cases [44], although similar trends were not as clearly observed in the untreated yields of leaf sheath and leaf internodes and indicate cell wall recalcitrance increases with maturity in stem only.

NaOH-pretreated leaf sheaths again showed significantly different trends compared to stem, with a trend of increasing hydrolysis yields with increasing internode in both the 6-h and 48-h hydrolysis yield cases. Leaf hydrolysis yields became similar between internodes in the 48-h yields for the NaOH-pretreated case, contrasting the general trend of decreasing yields with increasing internode seen in the untreated and 6-h hydrolysis yield for the NaOH-pretreated cases. From these observations, the application of alkaline pretreatment resulted in a change in recalcitrance between internodes in both leaf sheath and leaf fractions, although differences in the 6- and 48-h hydrolysis yield trends in leaf internodes suggest pretreatment also had an effect on hydrolysis rate as well. The contributions of composition and cell wall biopolymer structure to these differences in recalcitrance are described in the subsequent sections.

### Cell wall composition

**Fig. 3:**
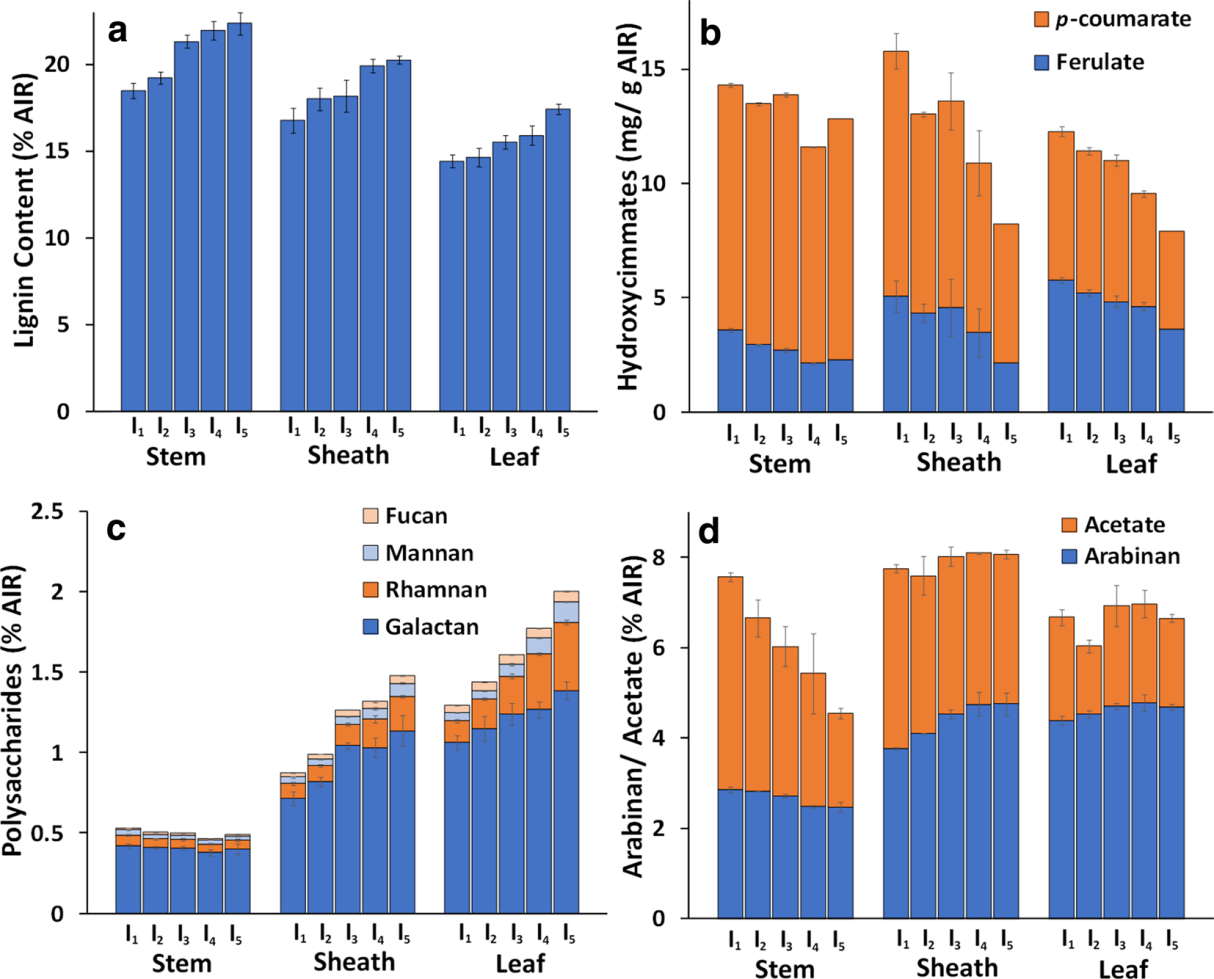
Compositional highlights of (**a**) lignin (**b**) hydroxycinnamates (**c**) minor polysaccharides, and (**d**) acetate and arabinan in switchgrass fractions with respect to internode.  Internodes I_1_–I_5_ are displayed in ascending order from *left* to *right* for each switchgrass anatomical fraction. All composition values were quantified on an extractives-free basis (AIR). Replicates (*n* = 3) displayed as average with standard deviations

The content of major cell wall biopolymers (cellulose, xylan, lignin), minor hemicelluloses (non-cellulosic glucan) and minor hemicellulose sugars (Rha, Ara, Man, Gal, Fuc), alkali-labile hydroxycinnamic acids (*p*-coumarate, ferulate), and lignin composition (syringyl and guaiacyl monomer yields by thioacidolysis) was quantified for stem, sheath, and leaf internodes, respectively. Key results that highlight observed trends found are presented in Fig. 3, while the complete dataset can be found in Additional file 1: Table S1.

The Klason lignin content demonstrated a clear increase with respect to internode for stem, sheath, and leaf (Fig. 3a), with results presumably corresponding to increasing maturation and development of secondary cell wall tissues following cell elongation and differentiation in older fractions. Overall, stem lignin content was higher compared to sheath and leaf, which is expected due to the abundance of sclerenchyma and xylem cells providing structural support for both upright growth and normal vascular transport [5]. It should be noted that ash was not quantified independently in this study, and may represent a portion of the quantified lignin [45]. However, ash content has been shown to be higher in abundance in leaf fractions when compared to stem and sheath fractions [43], likely limiting ash impact on trends observed in this study.

Alkali-labile hydroxycinnamic acids also varied significantly with internode, with both *p*CA and FA content decreasing between internodes I_1_–I_5_ for all switchgrass anatomical fractions (Fig. 3b). Decreases in *p*CA content with increasing internode maturity ranged from a 19% decrease from I_1_ to I_5_ in stem samples, to 53 and 45% in sheath and leaf samples, respectively. FA content also demonstrated the same trend between internodes I_1_–I_5_ with decreases of 47, 65, and 48% for stem, sheath, and leaf, respectively. Combined with the trend for lignin content (Fig. 3a), the inverse relationship between FA content and lignin content suggests that crosslinking of cell wall biopolymers via ferulates is an important feature of cell wall organization in tissues with less secondary cell wall lignification [18]. Prior work has concluded that total FA content decreases with increasing maturity (based on harvest date) concurrent with increasing lignin content in switchgrass [45], and is in agreement with the results of the present work in which maturity is based on internode rather than harvest date.

Composition analysis of polysaccharide content revealed a similar content of xylan between internodes (average content of 24.4% for stem and sheath and 19.8% for leaf), while galactan, mannan, rhamnan, and fucan, representing minor hemicellulose and structural pectin polysaccharides were higher in sheath and leaf fractions compared to stem and showed clear trends of increased abundance with tissue maturity in sheath and leaf internodes (Fig. 3c).

Functionally, this indicates in maturing stem internodes, pectic and minor hemicellulosic polysaccharides represent a smaller fraction of total cell wall polysaccharides, and the decreases in total compositional fraction suggest minimal continued synthesis of these polysaccharides during tissue maturation. Conversely, increased overall composition content in more mature sheath and leaf internodes suggest continued synthesis and accumulation of minor polysaccharides during maturation. The exception to this trend, non-cellulosic glucan, showed an increase with respect to internode in all three anatomical fractions as well as similar overall content of non-cellulosic glucan (2.5% overall for all samples), indicating increases in either β-glucan or xyloglucan content with internode maturation (Additional file 1: Table S1).

Stem arabinan content, indicative of glucuronoarabinoxylan (GAX), arabinogalactan (AG), and rhamnogalacturonan I (RG I) pectin side chains decreased slightly in stem internodes, while increasing only in leaf sheaths (Fig. 3d). Given the small decrease in overall xylan content between internodes, it can be hypothesized that increases in arabinan content correspond to an increase in GAX or RG I side chain abundance in leaf sheath cell walls [23]. Many cell wall polysaccharides are known to be O-acetylated, including pectins, xyloglucans, and xylans. Cell wall acetyl content (Fig. 3d) decreased significantly with stem internode maturity, with only minor decreases observed with respect to sheath and leaf internode maturity, indicating similarity in acetylated cell wall components between sheath and leaf internodes compared to stem.

### Non-cellulosic glucose and extractable sugar content

The accumulation of soluble sugars in the stems of grasses is of paramount importance to agriculturally important crops such as sugarcane (*Saccharum* spp.) and sweet sorghum (*Sorghum bicolor*). Soluble sugar abundance in other grasses used as feedstocks for cellulosic biofuels can be a significant fraction of total dry weight [50], with both genetic and environmental drivers responsible for starch and extractable sugar accumulation [46]. For example, soluble sugar synthesis in plants has been shown to be environment responsive, with significant accumulation of soluble sugars accompanying reduced structural carbohydrate and lignin content in switchgrass harvests subjected to drought conditions [47].

Notably, these sugars are often overlooked in many biomass-to-fuels studies or analyses and can present both processing and analytical challenges. Soluble sugars may mask structural polysaccharides in cell walls [48] resulting in false positives for carbohydrate content in screening techniques relating feedstock digestibility to biomass physical characteristics or high throughput digestibility studies that do not remove soluble sugars and starches before analysis [49]. As a processing challenge, contents of extractable sugars and starch can be degraded during many pretreatments (e.g., dilute acid or AFEX) representing a potentially significant loss of value sugars. Furthermore, sugar degradation products can present additional challenges to the biological conversion of sugars to biofuels or bioproducts. For example, in recent work using AFEX pretreatment on switchgrass subjected to drought environmental stress, imidazoles and pyrazines were formed by the reaction of ammonia with soluble sugars in high enough concentrations to significantly inhibit fermentation compared to switchgrass harvests not subjected to drought [47].

**Fig. 4:**
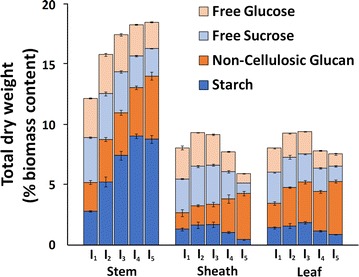
Native switchgrass non-cellulosic sugar composition with respect to internode. Internodes I_1_–I_5_ are displayed in ascending order from *left* to *right* for each switchgrass anatomical fraction. Compositions are represented as a percent of total dry weight. Replicates (*n* = 3) displayed as average with standard deviations

In the present work, non-cellulosic glucan (presumably as xyloglucan and β-glucan) and extractable such as sucrose, fructose, free glucose, and starches sugar contents were determined (Fig. 4) and represent a non-trivial fraction of overall feedstock mass. Stem internodes were observed to have significantly more soluble sugars compared to similar sheath and leaf internode contents, with soluble sugars combined with non-cellulosic glucan constituting between 12 and 18% of total dry weight. Starch content was dramatically different in stem internodes, accumulating to up to 8% of total biomass dry weight in internode I_5_. Comparatively, sheath and leaf starch content accounted for up to 2% dry weight, and decreased with internode maturity. One likely reason for such a high stem extractible sugar content was due to sample harvesting during grain filling [50].

Non-cellulosic glucan, presumably in the form of xyloglucans, β-glucans, and glucomannans, were observed to accumulate from internode I_1_ through I_5_ for all three anatomical fractions. Although comprising a small percentage of total biomass, β-glucans are generally more easily digested compared to cellulose, while xyloglucans may be closely associated to cellulose in the primary cell wall [51]. It can also be observed that the differences in the non-cellulosic glucan can be correlated to differences in most of the minor hemicellulose and pectin sugars (i.e., Fuc, Rha, Man, Gal as presented in Fig. 3c).

### Cell wall autofluorescence

**Fig. 5:**
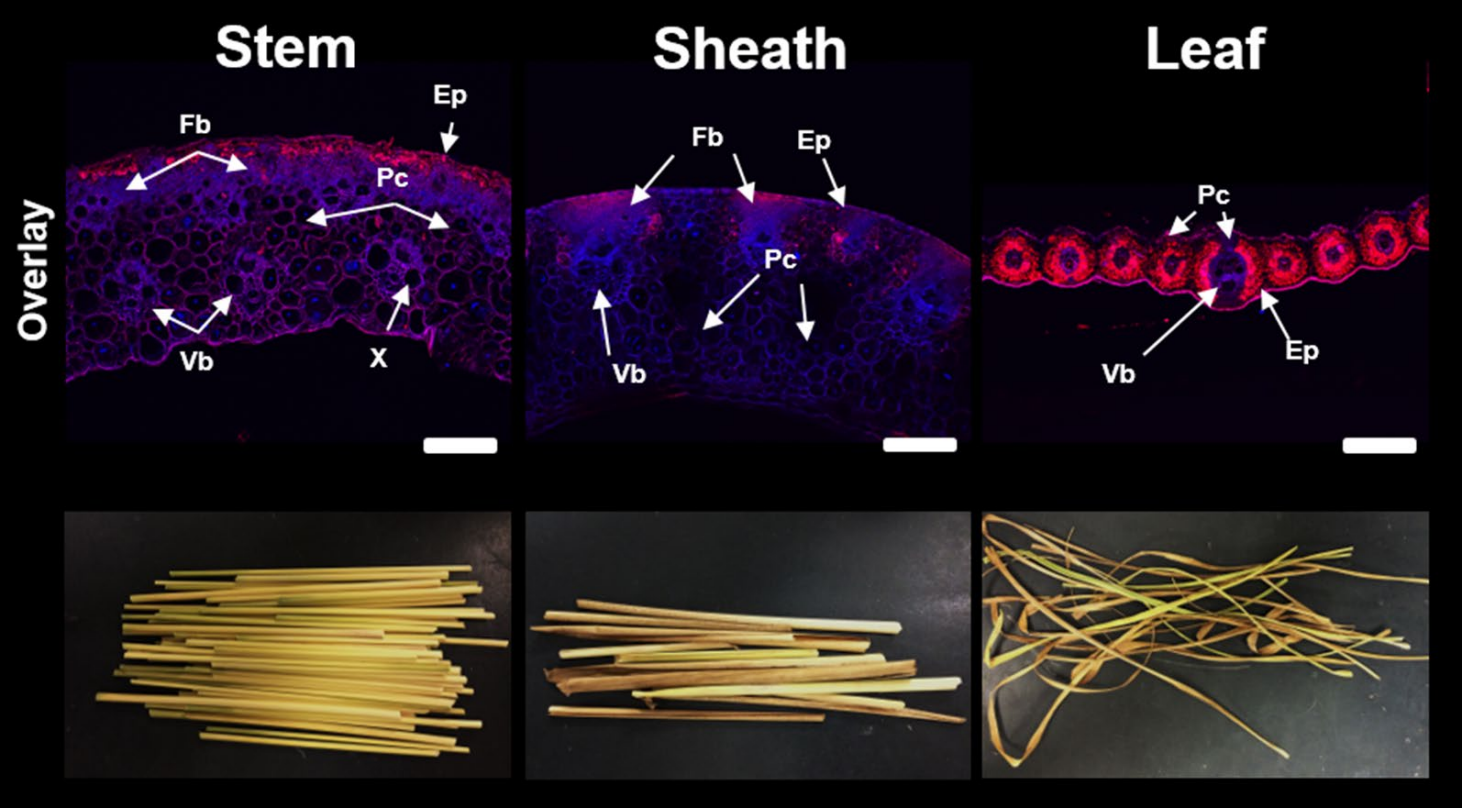
Examples of differences in macroscopic morphology, cell wall organization, and aromatic abundance as shown by autofluorescence between switchgrass anatomical fractions from internode I_2_. Confocal laser scanning microscopy carried out using laser emission wavelengths of 405 and 543 nm. Tissue types are denoted from the following notation: *Ep* epidermis, *Fb* fiber bundles, *Vb* vascular bundles, *Ph* phloem, *X* xylem, *Pc* parenchyma cells. *Scale bars* represent 200 µm

At both the macroscopic and microscopic scales as shown in Fig. 5, switchgrass stem, sheath, and leaf exhibit obvious significant differences in overall morphology, tissue organization, and cell type abundance. Structural (and compositional) heterogeneity across fractions has important implications for biomass recalcitrance. As one example, sclerenchyma secondary cell wall types in stems have significantly thicker cell walls and contain a high ratio of lignified secondary cell walls compared to partially lignified parenchyma type cells [45], resulting in anatomical fractions containing high contents of sclerenchyma cells expecting to have higher recalcitrance compared to parenchyma rich fractions.

**Fig. 6:**
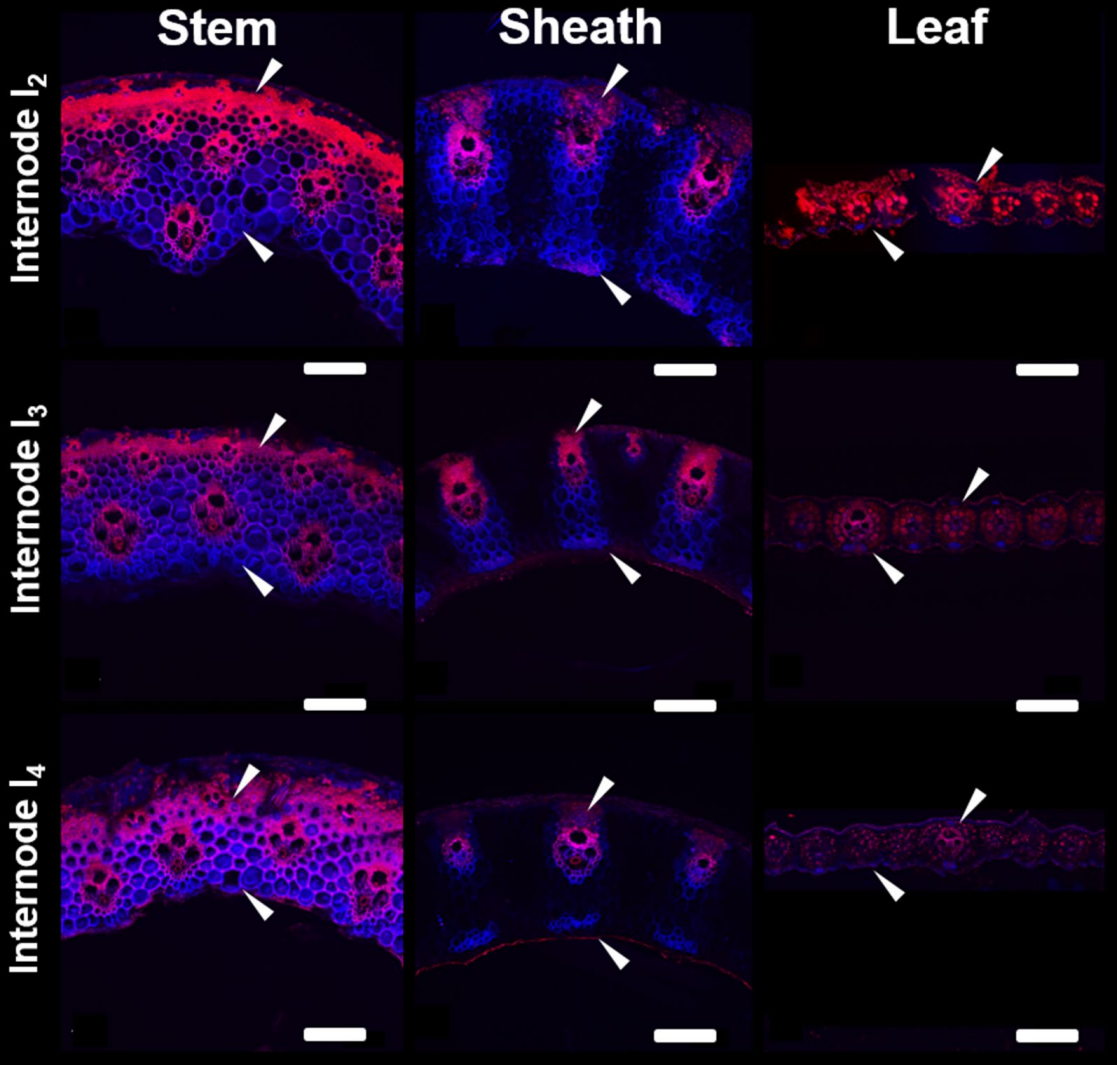
Cell wall autofluorescence cross-sections of NaOH-pretreated switchgrass anatomical fractions from internodes I_2_, I_3_, and I_4_. Confocal laser scanning microscopy carried out using an overlay of laser excitation wavelengths of 405 and 543 nm. *Scale bars* represent 200 µm

Analysis of cell wall phenolics using confocal microscopy often employs combinations of fluorescence tagging, chemical labels, and autofluorescence techniques to characterize the cell wall landscape at the micron-scale resolution [52, 53]. Using phenolic autofluorescence alone, general trends in phenolic localization and abundance can be obtained; however, the wide range of emission spectrums of phenolic compounds can limit deconvolution of specific phenolic components [54–56]. With a low-field excitation using a diode laser at 405 nm, hydroxycinnamates have an emission spectrum of around 420–460 nm (shown and denoted as blue) along with alkaloids, flavonoids, and other phenylpropanoids [56]. Using a helium neon laser excitation at 543 nm, most phenolics fluoresce with an emission spectrum of 600–650 nm (shown and denoted as red), however hydroxycinnamates do not [55]. One convoluting factor in this technique resides in the autofluorescence of chlorophyll. With an emission spectrum in the 600–700 nm region [56], chlorophyll will certainly provide signal contribution to the red emission spectrum from chlorophyll rich tissues such as epidermal and bundle sheath regions of leaf (Figs. 5, 6).

Overlay of both autofluorescence channels of whole biomass cross sections revealed several noticeable features of each organ (Fig. 5). Stem and sheath cross sections displayed similar architecture, with atactostele patterned vascular bundles, distinct regions of fiber bundles, and similar overall thickness [57]. The only notable difference resided in distribution of fiber cells between epidermal located vascular bundles in the sheath cross section. Both displayed blue and red emission patterns in all tissue types; however, signal intensity was much stronger in secondary cell wall abundant regions such as sclerenchyma cell fiber bundles, and vascular bundles. Leaf cross-section architecture was significantly different, with vascular bundles surrounded by a bundle sheath of parenchyma cells, and a highly lignified lower midrib region. Leaf autofluorescence showed a blue emission pattern in the vascular bundles along with some epidermal tissues, while the red emission spectrum was localized to the lower midrib and bundle sheath, and was most likely due to chlorophyll autofluorescence.

The impact of NaOH pretreatment on autofluorescence signature was examined for multiple internodes to gauge both organ and internode response to pretreatment (Fig. 6). In stem and leaf sheath cross sections, the red emission spectrum was localized to secondary cell wall tissue such as vascular tissue and fiber bundles, while the blue emission spectrum was observed predominantly in parenchyma cells.

Comparing cross sections of stem internodes I_2_–I_4_, there was a clear increase of the blue emission spectrum in the fiber bundles, while in leaf sheaths cross sections sampled from internodes I_2_–I_4_, there was a clear decrease of the blue emission spectrum in the parenchyma cells. Signal intensity from the red emission spectrum decreased in fiber and vascular cells in both stem and leaf sheaths from internode I_2_–I_4_. In addition to changes in signal intensity, cell wall thickening was also observed in the parenchyma and interfascicular tissues of stem internode cross sections, corresponding to a maturation of tissues [18]. Similar thickening was not as apparent in sheath or leaf cross sections.

These results suggest that following NaOH pretreatment, components that fluoresced red in the stem cross sections were either preferentially extracted due to pretreatment, or were observed in lower abundance relative to other components in more mature internodes. This may also suggest that following alkaline pretreatment, there were regions of hydroxycinnamates that remained unextracted or were more recalcitrant in stem internodes, as evidenced by the change in ratio of blue emission to red emission spectrum. The loss of both emission spectrum signals with internode maturity in sheath cross sections suggests either the removal of both components with alkaline pretreatment, or the loss of accessibility of both components for autofluorescence.

From the perspective of secondary cell wall development, more mature internode cross sections may have hydroxycinnamates in all cell types, while in younger stem internode cross sections, potential locations of hydroxycinnamates might be restricted predominantly to developing parenchyma cells. In sheath cross sections, younger internodes had potential locations of hydroxycinnamates in all cell types, with the eventual observance of the blue emission spectrum only in the vascular bundle regions. Leaf internodes following NaOH pretreatment had a noticeable decrease in both emission signal intensities, with the blue emission spectrum localized to the outer parenchyma and epidermal cells, and the red emission spectrum localized to the bundle sheath and epidermal cells. Given the autofluorescence of chlorophyll in the red emission spectrum, the decrease in red signal can be largely attributed to either chlorophyll removal from alkaline extraction, or a decrease in chlorophyll in more mature leaf internodes following senescence.

### Glycome profiling

**Fig. 7:**
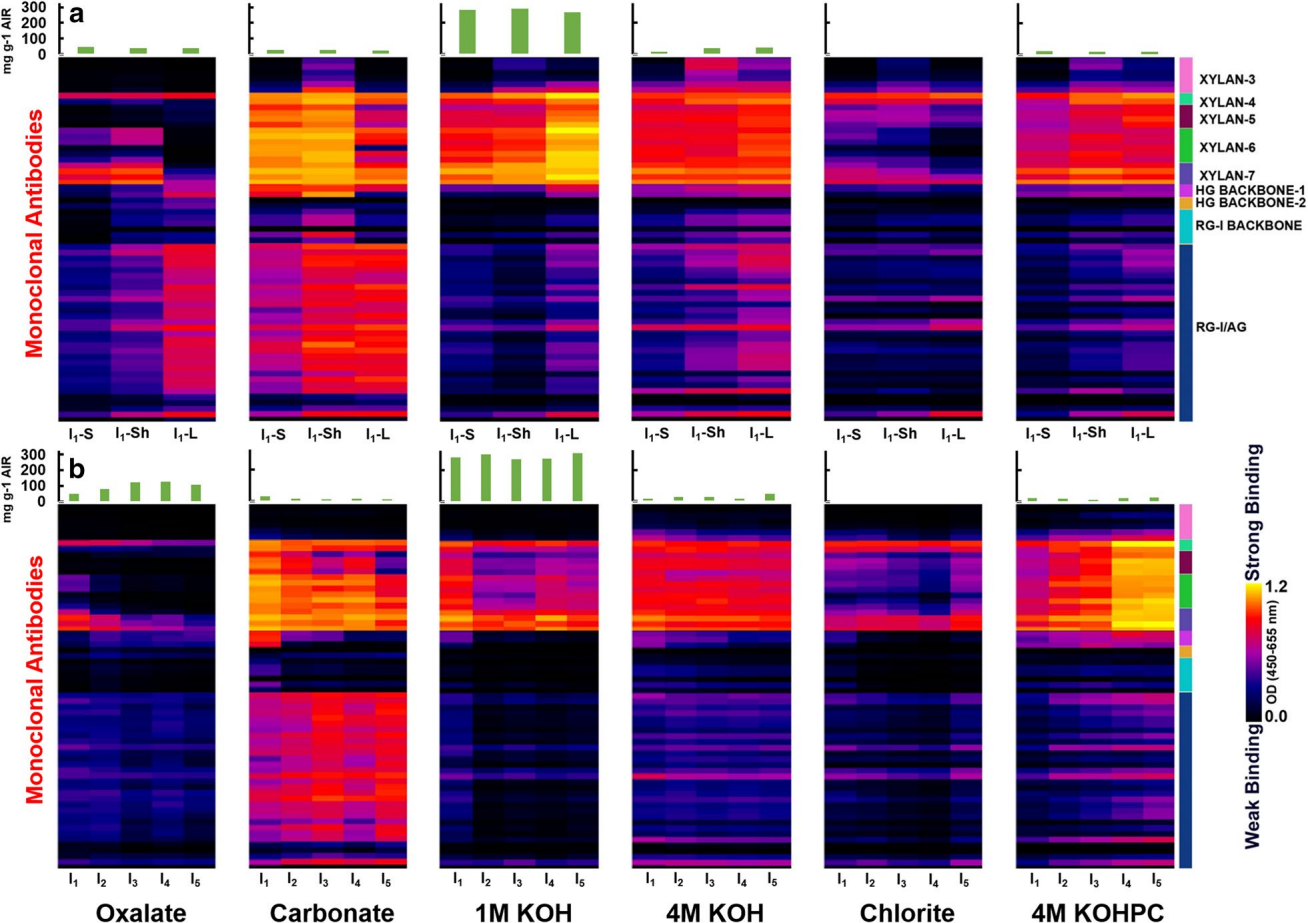
Abundance of select major non-cellulosic glycan epitopes in chemical extracts from stem, sheath, and leaf anatomical fractions of internodes I_1_ (**a**), and extracts from stem internodes I_1_–I_5_ (**b**). Full glycome profiles can be found in the Additional file 1. Extracts were screened by ELISA using a comprehensive suite of cell wall glycan-directed mAbs as described in "Methods". Binding response values are depicted as heat maps with *black*–*red*–*bright yellow* color scheme representing from no binding to strongest binding. The amount of carbohydrate material recovered per gram of AIR is depicted in the *bar graphs* (*green*) above the heat maps. The* panel* on the *right*-*hand side* of the heat map shows the groups of mAbs based on the class of cell wall glycan they each recognize

Glycome profiling was performed to assess organ-specific variations in the cell wall glycan composition and extractability across individual internodes of each anatomical fraction, I_1_-stem, I_1_-sheath, and I_1_-leaf. In Fig. 7a, selected regions of glycome profiles highlight abundancies of xylan and pectin epitopes in cell wall extracts, while the full glycome profile results can be found in Additional file 1: Figure S2.

With the oxalate extracts, extractability of xylan epitopes varied across anatomical fractions. For instance, both I_1_-stem and I_1_-sheath exhibited similar xylan epitope extractability patterns with significant abundance of arabinosylated xylan epitopes recognized by mAb CCRC-M154 of xylan-4 clade [58], unsubstituted xylan epitopes recognized by several mAbs from xylan-6 clade, and unsubstituted xylan epitopes recognized by all mAbs from xylan-7 clades. The I_1_-leaf oxalate extract was significantly different, showing an abundance of only arabinosylated xylan epitopes recognized by mAb CCRC-M154 of xylan-4 clade along with significantly reduced abundance of unsubstituted xylan epitopes recognized by mAbs from xylan-7 clades. Differences across anatomical fractions were also observed in the carbonate extract, with all three carbonate extracts containing most classes of xylan epitopes from unsubstituted and substituted regions. The I_1_-leaf sample again exhibited differences in the pattern of extractability with a marginal reduction in the overall abundancies of these epitopes, namely the Me-GlcA substituted xylan epitopes (xylan-5) and complete absence of xylan-6 epitopes. Xylan-3 epitopes were observed only in the I_1_-sheath, while all xylan clades were observed in the I_1_-stem extract.

Likely due to grass susceptibility to alkaline pretreatment [38], the 1 M KOH extract showed the largest portion of total carbohydrates released, with significant xylan epitope abundance in all three sample extracts. I_1_-leaf exhibited the strongest binding intensity for xylan epitopes 4 through 7 in the 1 M KOH extract, and this trend was mirrored in the 4 M KOH fraction. Similar patterns of xylan epitopes composition and extractability were recognized in the 4 M KOH extract; however, at this point, binding intensities across samples were largely similar and were probably the residual alkaline susceptible xylans that remained after the 1 M KOH extract [42]. The chlorite and subsequent 4 M KOH PC extracts were used to determine carbohydrates more tightly bound with lignin. Interestingly, the chlorite extract showed overall similar epitope recognition patterns for all three extracts as the oxalate extract, with the I_1_-leaf containing only xylan-4 and xylan-7 epitopes. Subsequent 4 M KOH PC extraction showed similar overall epitope extractability patterns and binding intensity as the 1 M KOH extract, with I_1_-sheath and I_1_-leaf extracts having the most intense binding for xylan-4 to xylan-7 epitopes.

From these results, it can be hypothesized that xylan integration within the cell walls of each anatomical fraction are not equivalent. Specifically, xylan association in the leaf fraction appeared to be more tightly integrated within the cell wall, as leaf xylan required a more severe basic 1 M KOH extract to observe significant xylan epitopes. Recognition of xylan-5 epitopes in the carbonate and 1 M KOH fraction was unexpected, as more substituted xylans such as arabinoxylan are thought to exist in primary cell walls, while less substituted and branched xylans are associated with the secondary cell wall [59]. This result may indicate an abundance in arabinosylation of leaf xylans compared to stem xylan in these switchgrass fractions [60]. Furthermore, higher xylan epitope recognition in the 4 M KOH PC suggests that xylan is more integrated with lignin in the leaf fraction, which may be a result of hemicelluloses in the leaf contributing more towards leaf internode recalcitrance compared to higher lignin containing fractions such as stem and sheath.

In terms of pectin epitope trends, while I_1_-stem and I_1_-sheath displayed marginal pectin epitope abundancies in the oxalate extract, the majority of recognized epitopes and highest abundancies was in the I_1_-leaf extract. Specifically, epitopes of the homogalacturonan backbone (HG backbone 1 and 2) and pectic arabinogalactan epitopes (RG-I/AG) were broadly recognized in the I_1_-leaf extract, with weak to moderate binding also occurring in the I_1_-sheath extract. More intense binding and recognition for the pectic arabinogalactan epitopes were observed in the carbonate extract for I_1_-sheath and I_1_-leaf, with a moderate amount of recognition in the I_1_-stem extract. In addition, HG 1 epitopes were observed in high intensity for all three extracts, and rhamnogalacturonan-I backbone (RG-I backbone) was recognized highest in the I_1_-sheath extract. Overall, a higher abundance of pectin backbone and pectic arabinogalactan (AG) epitopes was noted in I_1_-leaf samples in 1 M, 4 M KOH, and 4 M KOH PC extracts, while patterns in chlorite extracts were largely similar across samples.

From the pectin epitope recognition patterns, it is clear the stem anatomical fraction generally had lower overall pectin content that was also less recalcitrant. Sheath and leaf anatomical fractions displayed similar epitope recognition, suggesting similar pectin structures; however, differential epitope binding intensity suggests that each anatomical fraction has a unique organization of pectin in the cell wall. Pectic AGs are the major galactose containing pectin found in grasses [61], with the higher content of galactan found in the cell walls of leaf extracts suggesting higher overall pectic AGs content compared to stem samples [23].

### Glycome profiling for internode-specific extractability

Stem internodes I_1_–I_5_ were subjected to glycome profiling to examine trends in the non-cellulosic polysaccharide extractability and structure with respect to tissue maturity. As earlier, selected epitope profiles for xylan and pectins are shown in Fig. 7b, while the complete glycome profile can be found in Additional file 1: Figure S3.

Oxalate and carbonate extracts showed binding of xylan-4 and xylan-7 epitopes in all internodes, with the significantly enhanced appearance of xylan-6 epitopes in the carbonate fraction. There was a trend of reduced xylan-5 epitope abundance in the more mature internodes, corresponding to a decrease in extractable Me-GlcA substituted xylans in oxalate, carbonate, and 1 M KOH extracts. The 1 M KOH extract showed significant epitope extractability of xylan-4 and xylan-7, indicating that a large fraction of the total polysaccharides released in the 1 M KOH extracts were unsubstituted xylans or arabinoxylated xylans. The 4 M KOH fraction showed similar abundance of xylan-4, xylan-5, xylan-6, and xylan-7 epitopes among all developmental stages of internodes, while the 4 M KOH PC extracts showed an increasing trend of xylan mAb extractability in more mature internodes.

Taken together, these results indicate that internode maturity impacts xylan deconstruction and extractability. Specifically, higher abundance of xylans were recognized in less severe chemical extracts for younger internodes, while harsh extractions after chlorite delignification showed the opposite trend, indicating lignification plays a greater role in xylan recalcitrance in more mature internodes [62]. Pectin epitope abundance was largely observed in the carbonate and 4 M KOH PC extracts, with an increase in binding intensity of pectic AG epitopes with maturing internodes. In addition, a clear trend in decreasing binding strength was observed with the epitopes for the HG backbone 1, which is an indication of a transition from an abundance of primary cell walls to the development of secondary cell wall thickening [63]. In the 4 M KOH PC extracts, a similar trend was observed for the pectic AG as in the carbonate fraction; however, the HG backbone 1 epitope abundance increased with internode maturity. Pectin trends indicate that less severe extracts result in more extractable pectins from more mature internodes, which would indicate that pectins more closely associate within the cell wall in less mature internodes or are more easily liberated compared to xylans. At the same time, however, the trend observed post chlorite delignification suggests that there is a second fraction of pectins that are closely associated with lignin, as indicated by the increased pectin and pectin backbone abundance in more mature internodes. Pectin-lignin associations have been proposed in grasses in prior studies [23, 64].

## Discussion

**Fig. 8:**
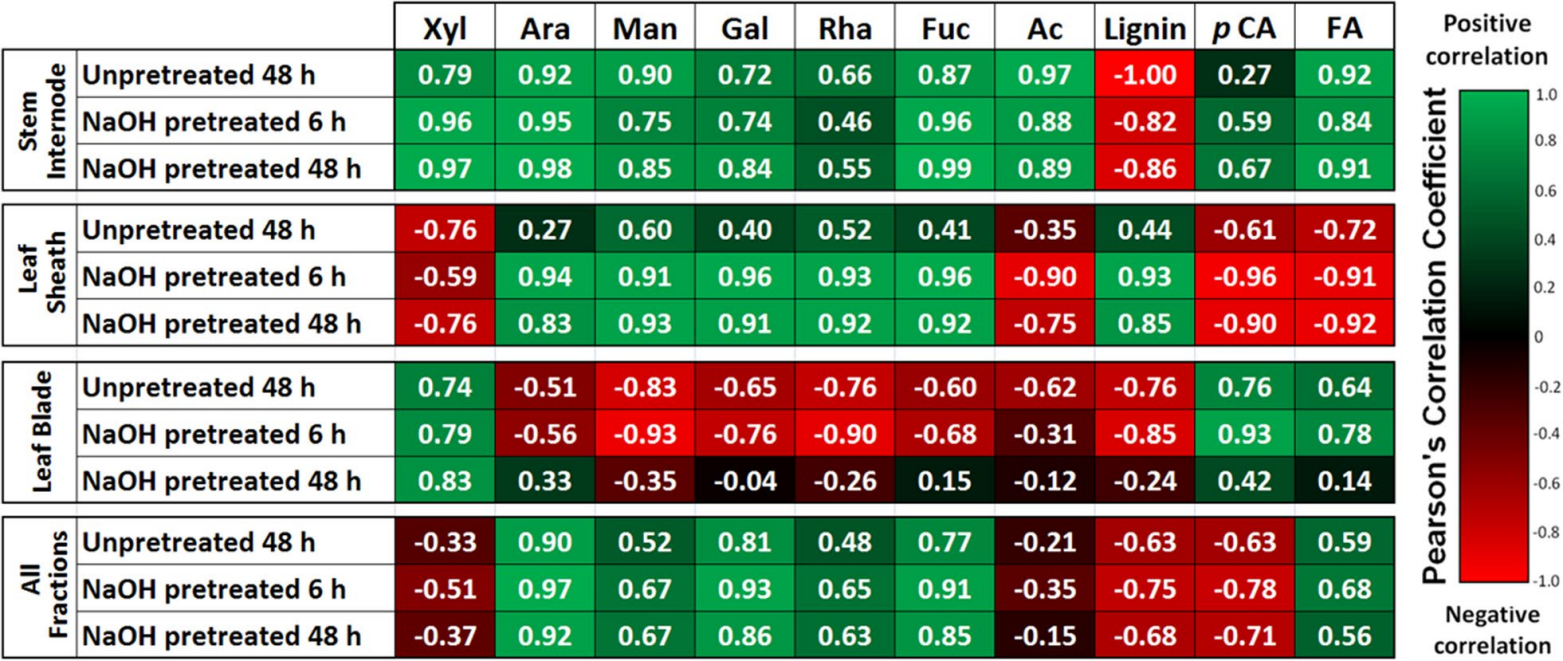
Summary of Pearson’s correlation coefficients between plant cell wall properties and glucose hydrolysis yields for each anatomical fraction and for pooled samples. Data demonstrate strong correlations between many properties and yields

**Fig. 9:**
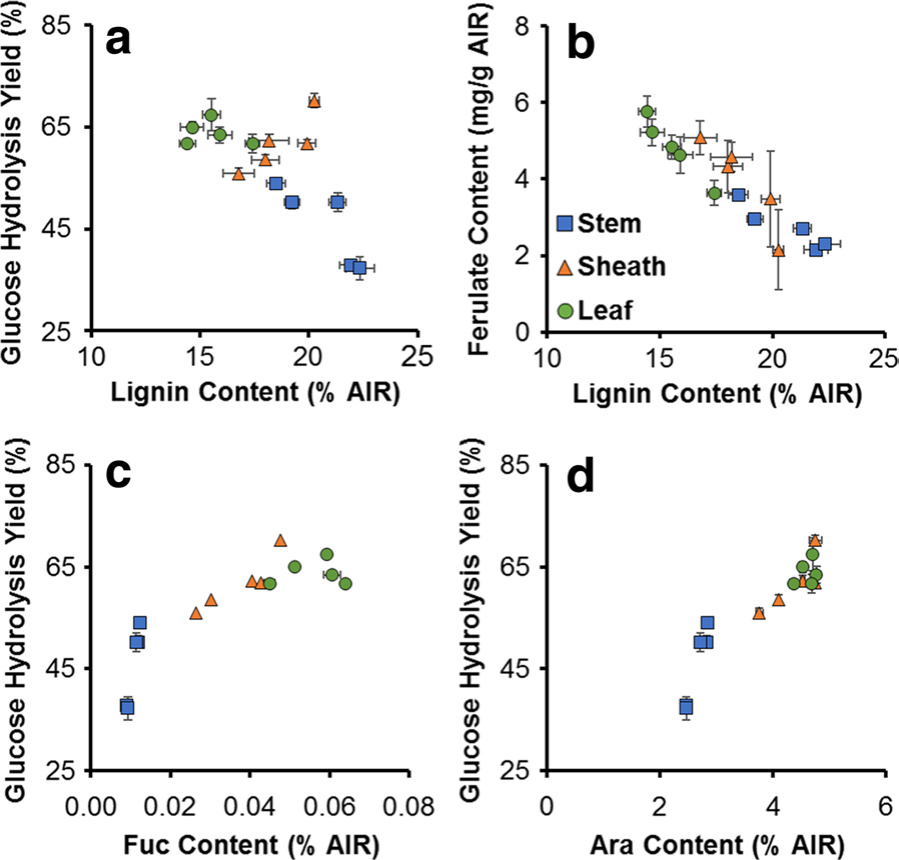
Select correlations between data relationships between either lignin content or ferulic acid against glucose hydrolysis yield represented as scatter plots. **a** 48-h glucose hydrolysis yields for NaOH-pretreat biomass versus lignin content, **b** ferulate content in relation to total lignin, and glucose hydrolysis yield with respect to **c** fucose and **d** arabinan content

Results for plant cell wall composition together with cell wall susceptibility to deconstruction by a coupled alkaline pretreatment and enzymatic hydrolysis demonstrate different switchgrass anatomical fractions possess significant differences both within fractions of varying maturity, and between different anatomical fractions. These trends are shown as a summary of Pearson’s correlation coefficients between cell wall properties and hydrolysis yields for either individual anatomical fractions or for all pooled fractions are presented in Fig. 8, while data from a few illustrative identified correlations are plotted in Fig. 9.

First, lignin content was found to exhibit a negative correlation with untreated and pretreated in stems and leaves (Fig. 8), while demonstrating a strong, significant negative correlation when all anatomical fractions are pooled for 48 h hydrolysis yields following pretreatment (*R* = −0.68; *p* value = 0.005; Figs. 8 and 9a). As similar trends are observed for untreated hydrolysis yields and pretreated hydrolysis yields, indicating the trend is related to anatomical fraction structure and maturation rather than pretreatment response. Stems contain the highest content of lignin of any of the anatomical fractions (Fig. 3a), with tissue maturity-dependent lignification and cell wall thickening [18] observed in confocal microscopy cross sections (Fig. 6). These results together suggest lignin is a defining feature of recalcitrance in the cell walls of developing stems. Notably, lignin is a well-known contributor to cell wall recalcitrance, and as one example, our previous work in diverse maize lines demonstrated that initial lignin content was a strong predictor of glucose hydrolysis yields for no pretreatment, while lignin content after mild NaOH pretreatment was a strong predictor of glucose hydrolysis yields comparable to what was performed in the present work [38]. Glycome profiling results do suggest that hemicelluloses may play a parallel role in inhibiting cell wall deconstruction, as a noted decrease in xylan epitope extractability was observed in maturing stem internodes until alkaline extraction post chlorite delignification (Fig. 7); however, higher lignification reducing xylan accessibility to extraction may have contributed to the observed trends.

Variability in lignin composition may also play a significant role in anatomical fraction-specific differences of recalcitrance. A strong negative correlation was observed between lignin and ferulate content for all fractions, shown in Fig. 9b (*R* ^2^ = 0.923; *p*-value = 10^−8^). Coupled with the observed decrease in ferulate content with increasing internodes shown in Fig. 3b and as reported previously in switchgrass stem internodes [18], suggesting that ferulate content within the cell wall is dependent upon both tissue maturity and lignin content. Ester crosslinking of cell wall biopolymers via ferulates is considered to be one of the major contributing features cell wall recalcitrance in low-lignin grass cell walls [7, 65]. Notably, ester crosslinks are susceptible to saponification during mild alkaline pretreatment relative to ether linkages between other monolignols, and may potentially contribute significantly to enzymatic hydrolysis yields following alkaline pretreatment.

Similar to stem internode composition, lignification increased in maturing leaf sheath cell walls; however, the positive correlation between 48-h glucose hydrolysis yields following NaOH pretreatment and lignin content in leaf sheath samples (Fig. 9a) suggests that other cell wall components, such as hemicellulose substitution, pectin abundance, and polysaccharide–lignin associations may be a more significant contributor to cell wall recalcitrance. In the leaf sheath, significant positive correlations were observed between minor polysaccharide sugars (Rha, Ara, Fuc, Gal, Man) and hydrolysis yields following pretreatment (Figs. 8, 9c, d), and considering the leaf sheath cell wall composition of each of these minor sugars can be more than double that in stem internodes (Fig. 3c), indicating these polymers may play a more important role in cell wall recalcitrance. Of note, arabinan, which is present as substitutions on the xylan backbone in GAX and to a substantially lesser extent as arabinogalactan (AG), is substantially higher in leaf sheath cell walls than in stem internodes and exhibits a strong positive correlation to glucose hydrolysis yields in pretreated biomass (Fig. 9d). The implications of this are that increased substitution of xylan with arabinosyl substitutions suggests a less tightly crosslinked cell wall matrix that may be more accessible to enzymatic deconstruction [21, 66]. This finding is in agreement with the work of Costa et al. [62], who found that the Ara:Xyl ratio in different anatomical fractions isolated from sugarcane stems was a strong predictor of enzymatic hydrolysis yields in un-pretreated biomass.

Although possessing similar arabinan content, the same trend was not observed in leaf internodes; lower cellulose, xylan, and lignin content in leaf internodes suggest that due to limited lignification in leaf internodes compared to stem internodes (Fig. 2), structural pectins and minor hemicellulose components with lower overall compositions playing a more significant role in cell wall recalcitrance. Results suggest that a relatively internode-independent, uniform response was observed in leaf cross-section phenolic autofluorescence to alkaline pretreatment (Fig. 6), corresponding to similar response to lignin removal. The effectiveness of enzymatic hydrolysis of leaf internodes following NaOH pretreatment further suggests that alkaline susceptible cell wall components did not have a differential effect upon leaf cell wall digestibility. Although displaying comparable yields to alkaline pretreated leaf sheaths, leaf cell walls may be more susceptible to a pretreatment that predominantly removes more polysaccharide components, such as liquid hot water pretreatment, which has shown to dramatically improve leaf digestibility [67].

Technologies for biomass fractionation or extraction employed either on-field or at a centralized processing facility offer the potential to simultaneously improve agronomic, logistics, and processing outcomes. As an example, on-site field fractionation of switchgrass could capitalize on more recalcitrant stem internodes residing at the bottom of the tiller, harvesting upper less mature internodes while leaving mature internodes on-field for soil coverage and nutrient retention. Given stem and sheath leaf fractions constitute half of leaf dry weight in prior reporting [43], loss of biomass yield would have to be weighed against increases in downstream sugar yields due to less recalcitrant materials representing a larger total fraction of biomass.

Additionally, grass stems can accumulate a substantial amount of soluble sugars in storage parenchyma cells surrounding the vascular tissues [68]. Given that soluble sugars can represent more than 10% of the switchgrass total dry weight in this study, water extraction may be proposed as a route to yield a stream rich in fermentable sugars. Sugarcane undergoes mechanical fractionation coupled with counter-current hot water extraction to remove soluble, fermentable sugars. Flow-through liquid hot water pretreatments are well-established pretreatment technologies [69]; counter-current processing configurations can be envisioned that integrate soluble sugar extraction with liquid hot water pretreatment, each taking place at their respective optimal temperatures.

## Conclusions

In this work, we were able to demonstrate that significant heterogeneity in cell wall structure, composition, properties within switchgrass results in substantially different feedstock response to processing in a cellulosic biorefinery. Feedstock heterogeneity may offer the potential to either tailor harvest approach, harvest time, or employ physical fractionation during processing to optimize processing outcomes such as sugar yield. Using switchgrass physically fractionated by anatomical fraction and internode, we identified compositional differences that impact recalcitrance and related results to macroscopic observations. Specifically, we showed that while all anatomical fractions experienced different extents of lignification between internodes, stem internode, leaf sheath, and leaf blade fractions were shown to have different structural features that dominate cell wall recalcitrance. Lignin content was identified as exhibiting a strong correlation to cell wall recalcitrance in stem internodes and initial recalcitrance in leaves. Leaf sheath and leaf cell wall recalcitrance were also shown to be impacted significantly by hemicellulose content and substitution, as well as structural pectin content. Ferulate and *p*-coumarate content were inversely correlated to lignin content in all fractions and decreased significantly with increasing internode maturity. Additionally, soluble sugars can account for a non-trivial fraction of total switchgrass dry weight, and represent a potential avenue to generate an enriched stream of easily extracted sugars to improve process economics.

## Additional file

**Additional file 1: Table S1.** Compiled AIR + destarched cell wall polysaccharides, acetate, and lignin content for five internodes of three switchgrass anatomical fractions. Samples are presented as an average (n = 3) with standard deviations following in additional Table S2. **Table S2.** Standard deviations for compiled AIR + destarched cell wall polysaccharides, acetate, and lignin content for five internodes of three switchgrass anatomical fractions. Samples are presented as an average (n = 3) with standard deviations following. **Figure S1.** Cell wall autofluorescence of switchgrass organ cross-sections from internode I_2_. Confocal laser scanning microscopy carried out using laser excitation wavelengths of 405 nm and 543 nm. Tissue types are denoted from the following notation: Ep – epidermis, Fb – fiber bundles, Vb – vascular bundles, Ph – phloem, X – xylem, Pc – Parenchyma cells. Scale bars represent 200 µm. **Figure S2.** Heat map abundance of major non-cellulosic glycan epitopes in oxalate, carbonate, 1 M KOH, 4 M KOH, chlorite, and 4 M KOH PC extracts from cell walls of stem, sheath, and leaf samples of internodes I_1_. Extracts were screened by ELISA using a comprehensive suite of cell wall glycan-directed mAbs as described in materials and methods. Binding response values are depicted as heat maps with black-red-bright yellow color scheme representing from no binding to strongest binding. The amount of carbohydrate material recovered per gram of AIR is depicted in the bar graphs (purple) above the heat maps. The panel on the right-hand side of the heat map shows the groups of mAbs based on the class of cell wall glycan they each recognize. **Figure S3.** Heat map abundance of major non-cellulosic glycan epitopes in oxalate, carbonate, 1 M KOH, 4 M KOH, chlorite, and 4 M KOHPC extracts from stem internodes I_1_-I_5_. Extracts were screened by ELISA using a comprehensive suite of cell wall glycan-directed mAbs as described in materials and methods. Binding response values are depicted as heat maps with black-red-bright yellow color scheme representing from no binding to strongest binding. The amount of carbohydrate material recovered per gram of AIR is depicted in the bar graphs (purple) above the heat maps. The panel on the right-hand side of the heat map shows the groups of mAbs based on the class of cell wall glycan they each recognize.

## Abbreviations

AG: arabinogalactan
AIR: alcohol insoluble residue
FA: ferulate
GAX: glucuronoarabinoxylan
HG: homogalacturonan
*p*CA: *p*-coumarate
RG: rhamnogalacturonan
XG: xyloglucan

## References

[1] Melillo JM, Richmond TC, Yohe GW (2014). Climate change impacts in the United States: The third national climate assessment.

[2] Tilman D, Socolow R, Foley JA, Hill J, Larson E, Lynd L, Pacala S, Reilly J, Searchinger T, Somerville C (2009). Beneficial biofuels-the food, energy, and environment trilemma. Science.

[3] U.S. Department of Energy. U.S. Billion-ton update: biomass supply for a bioenergy and bioproducts industry. In: Perlack D, Stokes BJ, editors. ORNL/TM-2011/224. Oak Ridge: Oak Ridge National Laboratory; 2011.

[4] Himmel ME, Ding SY, Johnson DK, Adney WS, Nimlos MR, Brady JW, Foust TD (2007). Biomass recalcitrance: engineering plants and enzymes for biofuels production. Science.

[5] Albersheim PD, Roberts K, Sederoff R, Staehelin A (2011). Plant cell walls: Garland Science.

[6] Pauly M, Gille S, Liu L, Mansoori N, Souza A, Schultink A, Xiong G (2013). Hemicellulose biosynthesis. Planta.

[7] Grabber JH, Ralph J, Lapierre C, Barrière Y (2004). Genetic and molecular basis of grass cell-wall degradability. I. Lignin–cell wall matrix interactions. C R Biol.

[8] Scheller HV, Ulvskov P (2010). Hemicelluloses. Annu Rev Plant Biol.

[9] Wilkerson CG, Mansfield SD, Lu F, Withers S, Park JY, Karlen SD, Gonzales-Vigil E, Padmakshan D, Unda F, Rencoret J (2014). Monolignol ferulate transferase introduces chemically labile linkages into the lignin backbone. Science.

[10] DeMartini JD, Pattathil S, Miller JS, Li HJ, Hahn MG, Wyman CE (2013). Investigating plant cell wall components that affect biomass recalcitrance in poplar and switchgrass. Energy Environ Sci.

[11] Lionetti V, Francocci F, Ferrari S, Volpi C, Bellincampi D, Galletti R, D’Ovidio R, De Lorenzo G, Cervone F (2010). Engineering the cell wall by reducing de-methyl-esterified homogalacturonan improves saccharification of plant tissues for bioconversion. Proc Natl Acad Sci USA.

[12] Cass CL, Lavell AA, Santoro N, Foster CE, Karlen SD, Smith RA, Ralph J, Garvin DF, Sedbrook JC (2016). Cell wall composition and biomass recalcitrance differences within a genotypically diverse set of *Brachypodium distachyon* inbred lines. Front Plant Sci..

[13] McLaughlin SB, Kszos LA (2005). Development of switchgrass (*Panicum virgatum*) as a bioenergy feedstock in the United States. Biomass Bioenerg.

[14] Somerville C, Youngs H, Taylor C, Davis SC, Long SP (2010). Feedstocks for lignocellulosic biofuels. Science.

[15] Parrish DJ, Casler MD, Monties A, Monties A (2012). The evolution of switchgrass as an energy crop. Switchgrass: a valuable biomass crop for energy.

[16] Bouton JH (2007). Molecular breeding of switchgrass for use as a biofuel crop. Curr Opin Genet Dev.

[17] Jung HG, Casler MD (2006). Maize stem tissues: cell wall concentration and composition during development. Crop Sci.

[18] Sarath G, Baird LM, Vogel KP, Mitchell RB (2007). Internode structure and cell wall composition in maturing tillers of switchgrass (*Panicum virgatum* L.). Biores Technol.

[19] Dien BS, Jung HJG, Vogel KP, Casler MD, Lamb JFS, Iten L, Mitchell RB, Sarath G (2006). Chemical composition and response to dilute-acid pretreatment and enzymatic saccharification of alfalfa, reed canarygrass, and switchgrass. Biomass Bioenerg.

[20] Sarath G, Mitchell RB, Sattler SE, Funnell D, Pedersen JF, Graybosch RA, Vogel KP (2008). Opportunities and roadblocks in utilizing forages and small grains for liquid fuels. J Ind Microbiol Biotechnol.

[21] Vogel KP, Casler MD, Dien BS (2016). Switchgrass biomass composition traits and their effects on its digestion by ruminants and bioconversion to ethanol. Crop Sci.

[22] Sarath G, Dien B, Saathoff AJ, Vogel KP, Mitchell RB, Chen H (2011). Ethanol yields and cell wall properties in divergently bred switchgrass genotypes. Biores Technol.

[23] da Costa RMF, Pattathil S, Avci U, Lee SJ, Hazen SP, Winters A, Hahn MG, Bosch M (2016). A cell wall reference profile for Miscanthus bioenergy crops highlights compositional and structural variations associated with development and organ origin. New Phytol.

[24] Bootsma JA, Shanks BH (2005). Hydrolysis characteristics of tissue fractions resulting from mechanical separation of corn stover. Appl Biochem Biotechnol.

[25] Crofcheck CL, Montross MD (2004). Effect of stover fraction on glucose production using enzymatic hydrolysis. Trans Am Soc Agric Eng.

[26] Montross MD, Crofcheck CL (2004). Effect of stover fraction and storage method on glucose production during enzymatic hydrolysis. Biores Technol.

[27] Hansey CN, Lorenz AJ, de Leon N (2010). Cell wall composition and ruminant digestibility of various maize tissues across development. Bioenerg Res..

[28] Ding SY, Liu YS, Zeng Y, Himmel ME, Baker JO, Bayer EA (2012). How does plant cell wall nanoscale architecture correlate with enzymatic digestibility?. Science.

[29] Hansen MA, Hidayat BJ, Mogensen KK, Jeppesen MD, Jørgensen B, Johansen KS, Thygesen LG (2013). Enzyme affinity to cell types in wheat straw (*Triticum aestivum* L.) before and after hydrothermal pretreatment. Biotechnol Biofuels.

[30] Zhang H, Fangel J, Willats WGT, Selig MJ, Lindedam J, Jorgensen H, Felby C (2014). Assessment of leaf/stem ratio in wheat straw feedstock and impact on enzymatic conversion. GCB Bioenerg.

[31] Chundawat SPS, Balan V, Dale BE (2007). Effect of particle size based separation of milled corn stover on AFEX pretreatment and enzymatic digestibility. Biotechnol Bioeng.

[32] Garlock RJ, Chundawat SPS, Balan V, Dale BE (2009). Optimizing harvest of corn stover fractions based on overall sugar yields following ammonia fiber expansion pretreatment and enzymatic hydrolysis. Biotechnol Biofuels.

[33] Foster CE, Martin TM, Pauly M (2010). Comprehensive compositional analysis of plant cell walls (lignocellulosic biomass) part II: carbohydrates. J Vis Exp.

[34] Sluiter JB, Ruiz RO, Scarlata CJ, Sluiter AD, Templeton DW (2010). Compositional analysis of lignocellulosic feedstocks. 1. Review and description of methods. J Agric Food Chem.

[35] Li M, Foster C, Kelkar S, Pu Y, Holmes D, Ragauskas A, Saffron C, Hodge D (2012). Structural characterization of alkaline hydrogen peroxide pretreated grasses exhibiting diverse lignin phenotypes. Biotechnol Biofuels.

[36] Foster CE, Martin TM, Pauly M (2010). Comprehensive compositional analysis of plant cell walls (lignocellulosic biomass) part I: lignin. J Vis Exp..

[37] Santoro N, Cantu SL, Tornqvist C-E, Falbel TG, Bolivar JL, Patterson SE, Pauly M, Walton JD (2010). A high-throughput platform for screening milligram quantities of plant biomass for lignocellulose digestibility. Bioenerg Res.

[38] Li M, Heckwolf M, Crowe JD, Williams DL, Magee TD, Kaeppler SM, de Leon N, Hodge DB (2015). Cell-wall properties contributing to improved deconstruction by alkaline pre-treatment and enzymatic hydrolysis in diverse maize (*Zea mays* L.) lines. J Exp Bot.

[39] DeMartini JD, Pattathil S, Avci U, Szekalski K, Mazumder K, Hahn MG, Wyman CE (2011). Application of monoclonal antibodies to investigate plant cell wall deconstruction for biofuels production. Energy Environ Sci.

[40] Pattathil S, Avci U, Miller JS, Hahn MG. Immunological approaches to plant cell wall and biomass characterization: glycome profiling. In: Biomass conversion. Berlin: Springer; 2012. p. 61–72. 10.1007/978-1-61779-956-3_6 22843389

[41] Pattathil S, Avci U, Baldwin D, Swennes AG, McGill JA, Popper Z, Bootten T, Albert A, Davis RH, Chennareddy C (2010). A comprehensive toolkit of plant cell wall glycan-directed monoclonal antibodies. Plant Physiol.

[42] Pattathil S, Hahn MG, Dale BE, Chundawat SP (2015). Insights into plant cell wall structure, architecture, and integrity using glycome profiling of native and AFEX™-pre-treated biomass. J Exp Bot.

[43] Hu ZJ, Foston M, Ragauskas AJ (2011). Comparative studies on hydrothermal pretreatment and enzymatic saccharification of leaves and internodes of alamo switchgrass. Biores Technol.

[44] Shen H, Fu CX, Xiao XR, Ray T, Tang YH, Wang ZY, Chen F (2009). Developmental control of lignification in stems of lowland switchgrass variety Alamo and the effects on saccharification efficiency. Bioenerg Res.

[45] Grabber J, Jung G, Hill R (1991). Chemical composition of parenchyma and sclerenchyma cell walls isolated from orchardgrass and switchgrass. Crop Sci.

[46] Chuck GS, Tobias C, Sun L, Kraemer F, Li C, Dibble D, Arora R, Bragg JN, Vogel JP, Singh S (2011). Overexpression of the maize Corngrass1 microRNA prevents flowering, improves digestibility, and increases starch content of switchgrass. Proc Natl Acad Sci USA.

[47] Ong RG, Higbee A, Bottoms S, Dickinson Q, Xie D, Smith SA, Serate J, Pohlmann E, Jones AD, Coon JJ (2016). Inhibition of microbial biofuel production in drought-stressed switchgrass hydrolysate. Biotechnol Biofuels.

[48] Garlock RJ, Balan V, Dale BE, Ramesh Pallapolu V, Lee YY, Kim Y, Mosier NS, Ladisch MR, Holtzapple MT, Falls M (2011). Comparative material balances around pretreatment technologies for the conversion of switchgrass to soluble sugars. Biores Technol.

[49] Decker SR, Carlile M, Selig MJ, Doeppke C, Davis M, Sykes R, Turner G, Ziebell A, Himmel ME (2012). Reducing the effect of variable starch levels in biomass recalcitrance screening. Biomass conversion: methods and protocols, methods in molecular biology.

[50] Slewinski TL (2012). Non-structural carbohydrate partitioning in grass stems: a target to increase yield stability, stress tolerance, and biofuel production. J Exp Bot.

[51] Park YB, Cosgrove DJ (2012). A revised architecture of primary cell walls based on biomechanical changes induced by substrate-specific endoglucanases. Plant Physiol.

[52] Tobimatsu Y, Wagner A, Donaldson L, Mitra P, Niculaes C, Dima O, Kim JI, Anderson N, Loque D, Boerjan W (2013). Visualization of plant cell wall lignification using fluorescence-tagged monolignols. Plant J.

[53] O’brien T, Feder N, McCully ME (1964). Polychromatic staining of plant cell walls by toluidine blue. Protoplasma.

[54] Donaldson L (2013). Softwood and hardwood lignin fluorescence spectra of wood cell walls in different mounting media. IAWA J.

[55] García-Plazaola JI, Fernández-Marín B, Duke SO, Hernández A, López-Arbeloa F, Becerril JM (2015). Autofluorescence: biological functions and technical applications. Plant Sci.

[56] Hutzler P, Fischbach R, Heller W, Jungblut TP, Reuber S, Schmitz R, Veit M, Weissenböck G, Schnitzler J-P (1998). Tissue localization of phenolic compounds in plants by confocal laser scanning microscopy. J Exp Bot.

[57] Matos DA, Whitney IP, Harrington MJ, Hazen SP (2013). Cell walls and the developmental anatomy of the *Brachypodium distachyon* stem internode. PLoS ONE.

[58] Schmidt D, Schuhmacher F, Geissner A, Seeberger PH, Pfrengle F (2015). Automated synthesis of arabinoxylan-oligosaccharides enables characterization of antibodies that recognize plant cell wall glycans. Chem Eur J.

[59] Suzuki K, Kitamura S, Kato Y, Itoh T (2000). Highly substituted glucuronoarabinoxylans (hsGAXs) and low-branched xylans show a distinct localization pattern in the tissues of *Zea mays* L.. Plant Cell Physiol.

[60] de Souza AP, Leite DCC, Pattathil S, Hahn MG, Buckeridge MS (2013). Composition and structure of sugarcane cell wall polysaccharides: implications for second-generation bioethanol production. Bioenerg Res.

[61] Mohnen D (2008). Pectin structure and biosynthesis. Curr Opin Plant Biol.

[62] Costa THF, Vega-Sanchez ME, Milagres AMF, Scheller HV, Ferraz A (2016). Tissue-specific distribution of hemicelluloses in six different sugarcane hybrids as related to cell wall recalcitrance. Biotechnol Biofuels.

[63] Cosgrove DJ (2005). Growth of the plant cell wall. Nat Rev Mol Cell Biol.

[64] Shen H, Poovaiah CR, Ziebell A, Tschaplinski TJ, Pattathil S, Gjersing E, Engle NL, Katahira R, Pu Y, Sykes R (2013). Enhanced characteristics of genetically modified switchgrass (*Panicum virgatum* L.) for high biofuel production. Biotechnol Biofuels.

[65] Ralph J (2010). Hydroxycinnamates in lignification. Phytochem Rev.

[66] Busse-Wicher M, Grantham NJ, Lyczakowski JJ, Nikolovski N, Dupree P (2016). Xylan decoration patterns and the plant secondary cell wall molecular architecture. Biochem Soc Trans.

[67] Zeng M, Ximenes E, Ladisch MR, Mosier NS, Vermerris W, Huang C-P, Sherman DM (2012). Tissue-specific biomass recalcitrance in corn stover pretreated with liquid hot-water: enzymatic hydrolysis (part 1). Biotechnol Bioeng.

[68] Rae AL, Grof CP, Casu RE, Bonnett GD (2005). Sucrose accumulation in the sugarcane stem: pathways and control points for transport and compartmentation. Field Crops Res.

[69] Mosier N, Wyman C, Dale B, Elander R, Lee YY, Holtzapple M, Ladisch M (2005). Features of promising technologies for pretreatment of lignocellulosic biomass. Biores Technol.

