# Supplementary material for: Identification of developmental stage and anatomical fraction contributions to cell wall recalcitrance in switchgrass
Source: Biotechnol Biofuels. 2017 Jul 15;10:184. doi: 10.1186/s13068-017-0870-5 (PMC5512841; doi:10.1186/s13068-017-0870-5)
Supplement: Supplementary file 1 — Additional file 1: Table S1. Compiled AIR + destarched cell wall polysaccharides, acetate, and lignin content for five internodes of three switchgrass anatomical fractions. Samples are presented as an average (n = 3) with standard deviations following in additional Table S2. Table S2. Standard deviations for compiled AIR + destarched cell wall polysaccharides, acetate, and lignin content for five internodes of three switchgrass anatomical fractions. Samples are presented as an average (n = 3) with standard deviations following. Figure S1. Cell wall autofluorescence of switchgrass organ cross-sections from internode I2. Confocal laser scanning microscopy carried out using laser excitation wavelengths of 405 nm and 543 nm. Tissue types are denoted from the following notation: Ep – epidermis, Fb – fiber bundles, Vb – vascular bundles, Ph – phloem, X – xylem, Pc – Parenchyma cells. Scale bars represent 200 µm. Figure S2. Heat map abundance of major non-cellulosic glycan epitopes in oxalate, carbonate, 1 M KOH, 4 M KOH, chlorite, and 4 M KOH PC extracts from cell walls of stem, sheath, and leaf samples of internodes I1. Extracts were screened by ELISA using a comprehensive suite of cell wall glycan-directed mAbs as described in materials and methods. Binding response values are depicted as heat maps with black-red-bright yellow color scheme representing from no binding to strongest binding. The amount of carbohydrate material recovered per gram of AIR is depicted in the bar graphs (purple) above the heat maps. The panel on the right-hand side of the heat map shows the groups of mAbs based on the class of cell wall glycan they each recognize. Figure S3. Heat map abundance of major non-cellulosic glycan epitopes in oxalate, carbonate, 1 M KOH, 4 M KOH, chlorite, and 4 M KOHPC extracts from stem internodes I1-I5. Extracts were screened by ELISA using a comprehensive suite of cell wall glycan-directed mAbs as described in materials and methods. Binding response values are de [file 13068_2017_870_MOESM1_ESM.pdf]

	Stem					Sheath					Leaf				
	I_1	I_2	I_3	I_4	I_5	I_1	I_2	I_3	I_4	I_5	I_1	I_2	I_3	I_4	I_5
**Cellulose (% AIR)**	43.93	50.96	50.51	50.33	46.42	40.07	48.12	47.9	42.97	42.59	35.78	36.72	36.22	37.27	36.69
**Xylan (% AIR)**	25.41	25.56	25.25	22.79	23.09	24.39	25.37	25.03	24.59	22.78	19.59	20.33	20.37	19.86	18.79
**Arabinan (% AIR)**	2.85	2.83	2.72	2.47	2.47	3.76	4.1	4.53	4.75	4.75	4.38	4.53	4.71	4.77	4.69
**Mannan (% AIR)**	0.03	0.03	0.03	0.02	0.03	0.04	0.04	0.05	0.07	0.08	0.05	0.05	0.07	0.1	0.13
**Galactan (% AIR)**	0.42	0.41	0.41	0.38	0.4	0.72	0.82	1.04	1.03	1.14	1.06	1.15	1.24	1.27	1.38
**Rhamnan (% AIR)**	0.07	0.05	0.05	0.05	0.05	0.09	0.1	0.13	0.18	0.21	0.13	0.18	0.23	0.35	0.43
**Fucan (% AIR)**	0.01	0.01	0.01	0.01	0.01	0.03	0.03	0.04	0.04	0.05	0.04	0.05	0.06	0.06	0.06
**Non-cellulosic glucan (% AIR)**	1.8	2.06	2.68	3.02	3.05	1.92	2	2.36	2.74	2.81	2.31	2.39	2.64	3.22	3.68
**Acetate (% AIR)**	4.72	3.83	3.31	2.95	2.07	3.99	3.49	3.49	3.35	3.32	2.3	1.51	2.22	2.19	1.96
**Lignin (% AIR)**	18.49	19.23	21.33	21.94	22.35	16.78	18.01	18.18	19.92	20.26	14.43	14.65	15.53	15.91	17.42
***p*-coumaric acid yield (mg/g AIR)**	10.71	10.54	11.16	9.45	10.51	10.71	8.7	9.02	7.42	6.09	6.51	6.2	6.17	4.92	4.29
**Ferulic acid yield (mg/g AIR)**	3.59	2.95	2.71	2.15	2.3	5.08	4.33	4.58	3.48	2.15	5.77	5.22	4.84	4.63	3.64
**Syringyl (S) monomer yield (mg/g AIR)**	1.55	1.84	1.87	1.88	2.38	0.87	0.72	0.73	0.63	0.65	0.43	0.42	0.41	0.52	0.69
**Guaiacyl (G) monomer yield (mg/g AIR)**	3.2	3.59	3.55	3.78	4.76	2.48	2.23	2.13	2	2.09	1.33	1.26	1.24	1.5	2.13
***p*- hydroxyphenyl (H) monomer yield (mg/g AIR)**	0.15	0.16	0.14	0.16	0.17	0.1	0.09	0.1	0.09	0.1	0.08	0.08	0.07	0.09	0.1

Supplemental Table S1. Table of compiled AIR + destarched cell wall polysaccharides, acetate, and lignin content for five internodes of three switchgrass anatomical fractions. Samples are presented as an average (n = 3) with standard deviations following in **Supplemental Table S2**.

	Stem					Sheath					Leaf				
	I_1	I_2	I_3	I_4	I_5	I_1	I_2	I_3	I_4	I_5	I_1	I_2	I_3	I_4	I_5
**Cellulose (% AIR)**	1.71	4.87	3.32	4.34	5.26	6.02	4.41	4.77	5.51	2.03	1.19	1.87	2.59	0.80	4.43
**Xylan (% AIR)**	0.81	0.55	0.64	0.74	1.17	0.49	0.39	0.51	1.19	1.62	0.52	0.89	0.41	1.32	0.73
**Arabinan (% AIR)**	0.07	0.01	0.04	0.03	0.11	0.03	0.01	0.09	0.27	0.25	0.10	0.09	0.06	0.19	0.06
**Mannan (% AIR)**	0.00	0.00	0.00	0.00	0.00	0.01	0.00	0.00	0.01	0.01	0.00	0.00	0.01	0.00	0.00
**Galactan (% AIR)**	0.01	0.01	0.01	0.02	0.03	0.04	0.03	0.02	0.06	0.09	0.04	0.08	0.07	0.05	0.06
**Rhamnan (% AIR)**	0.01	0.01	0.01	0.00	0.01	0.01	0.01	0.01	0.02	0.01	0.01	0.01	0.02	0.01	0.02
**Fucan (% AIR)**	0.00	0.00	0.00	0.00	0.00	0.00	0.00	0.00	0.00	0.00	0.00	0.00	0.00	0.00	0.00
**Non-cellulosic glucan (% AIR)**	0.16	0.16	0.19	0.16	0.34	0.30	0.08	0.17	0.36	0.19	0.16	0.06	0.15	0.12	0.13
**Acetate (% AIR)**	0.09	0.41	0.45	0.90	0.11	0.09	0.42	0.22	0.02	0.10	0.18	0.14	0.45	0.30	0.09
**Lignin (% AIR)**	0.43	0.36	0.39	0.53	0.66	0.73	0.64	0.92	0.41	0.23	0.36	0.53	0.41	0.55	0.31
***p*-coumaric acid yield (mg/g AIR)**	0.04	0.10	0.07	0.09	0.01	0.53	0.77	0.11	1.25	1.41	0.25	0.21	0.17	0.25	0.14
**Ferulic acid yield (mg/g AIR)**	0.06	0.08	0.03	0.08	0.04	0.43	0.68	0.39	1.26	1.05	0.16	0.13	0.14	0.23	0.18
**Syringyl (S) monomer yield (mg/g AIR)**	0.09	0.13	0.07	0.11	0.07	0.06	0.05	0.05	0.02	0.05	0.01	0.01	0.03	0.03	0.02
**Guaiacyl (G) monomer yield (mg/g AIR)**	0.18	0.22	0.12	0.27	0.12	0.10	0.12	0.11	0.04	0.03	0.04	0.07	0.08	0.05	0.04
***p*- hydroxyphenyl (H) monomer yield (mg/g AIR)**	0.01	0.00	0.01	0.01	0.01	0.00	0.00	0.00	0.01	0.00	0.00	0.00	0.00	0.01	0.00

Supplemental Table S2. Table of standard deviations for compiled AIR + destarched cell wall polysaccharides, acetate, and lignin content for five internodes of three switchgrass anatomical fractions. Samples are presented as an average (n = 3) with standard deviations following.

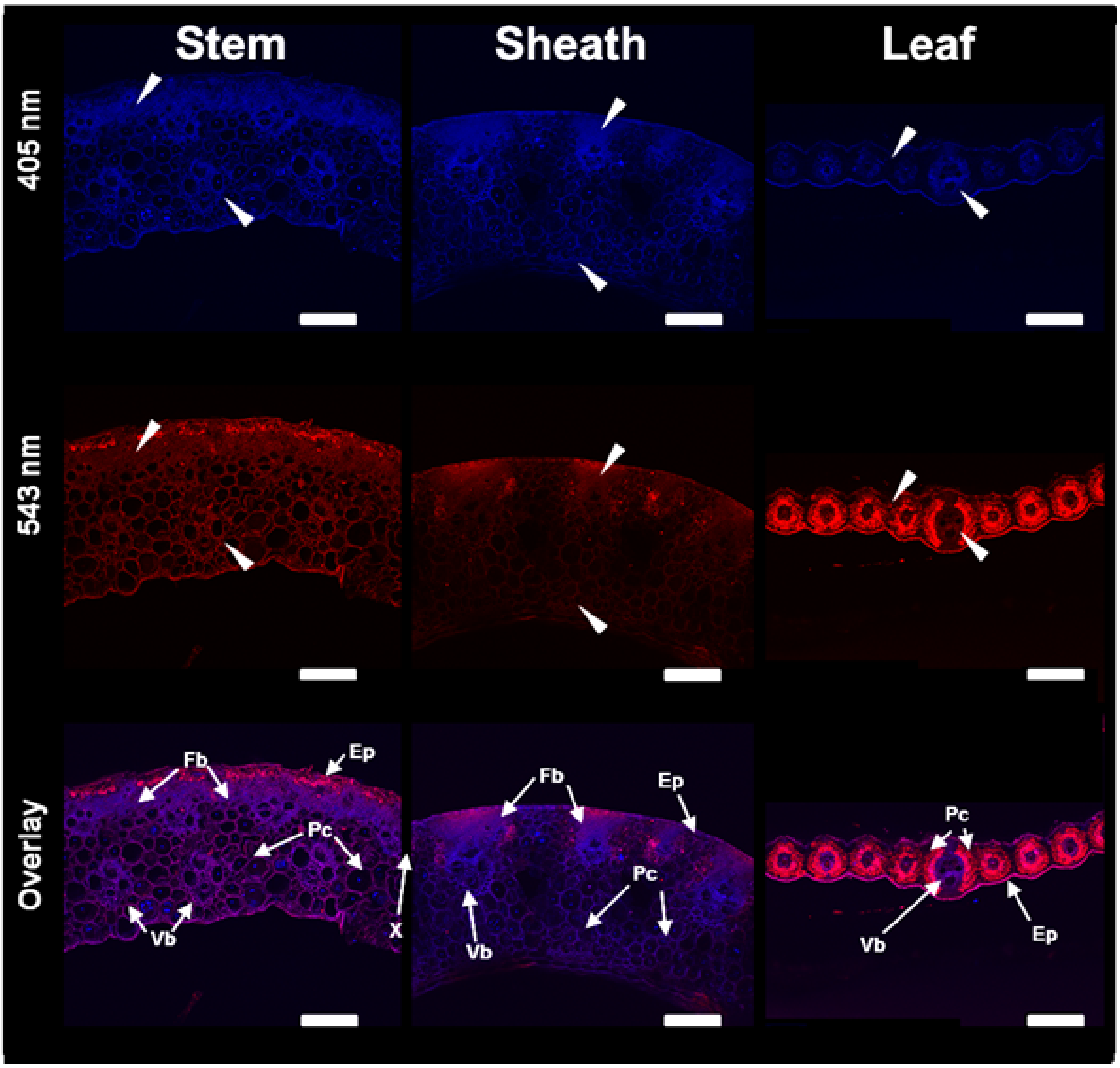

Supplemental Figure S1. Cell wall autofluorescence of switchgrass organ cross-sections from internode I_2. Confocal laser scanning microscopy carried out using laser excitation wavelengths of 405nm and 543nm. Tissue types are denoted from the following notation: Ep – epidermis, Fb – fiber bundles, Vb – vascular bundles, Ph – phloem, X – xylem, Pc – Parenchyma cells. Scale bars represent 200 µm.

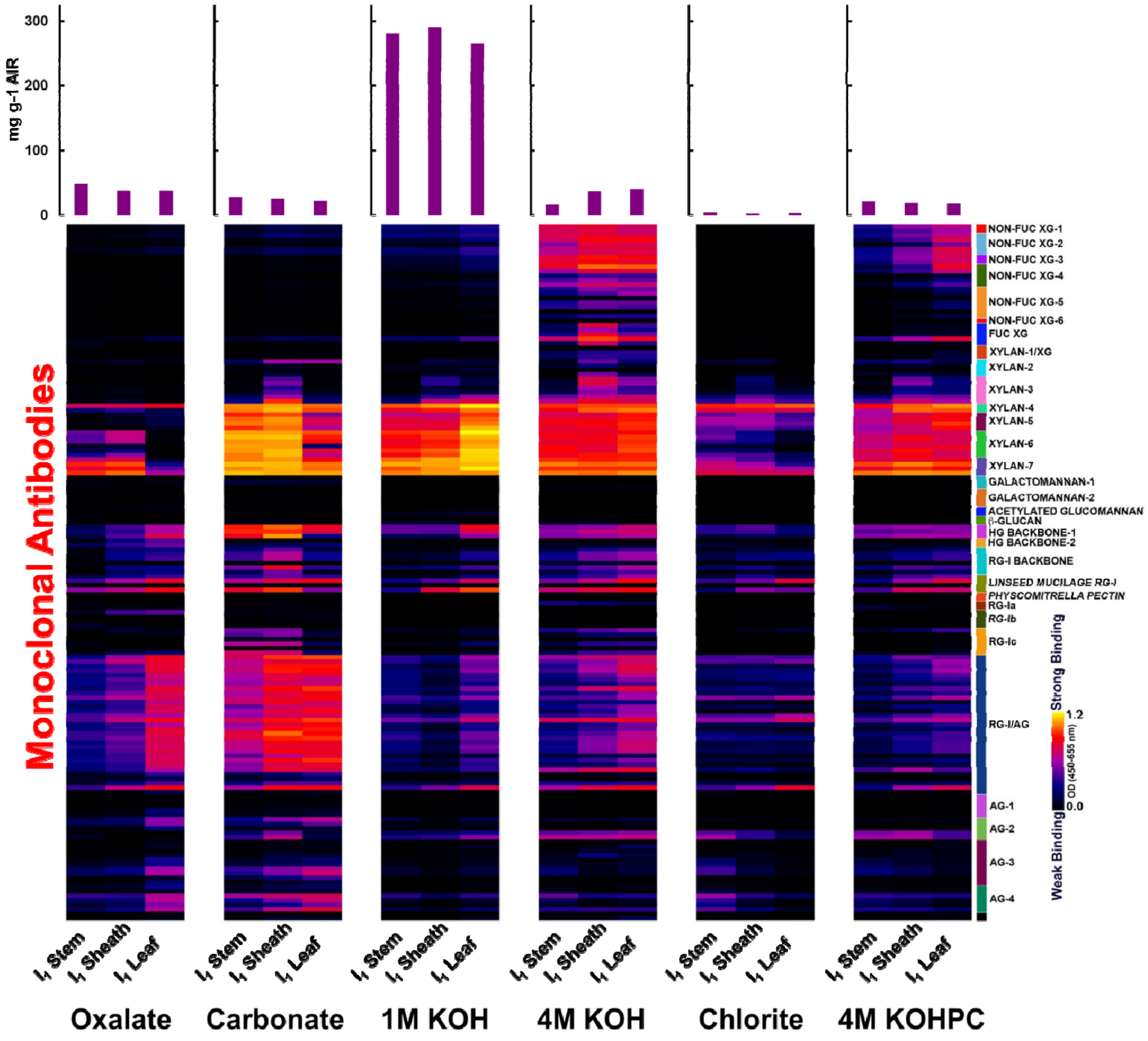

Supplemental Figure S2. Heat map abundance of major non-cellulosic glycan epitopes in oxalate, carbonate, 1M KOH, 4M KOH, chlorite, and 4M KOH PC extracts from cell walls of stem, sheath, and leaf samples of internodes I_1. Extracts were screened by ELISA using a comprehensive suite of cell wall glycan-directed mAbs as described in materials and methods. Binding response values are depicted as heat maps with black-red-bright yellow color scheme representing from no binding to strongest binding. The amount of carbohydrate material recovered per gram of AIR is depicted in the bar graphs (purple) above the heat maps. The panel on the right-hand side of the heat map shows the groups of mAbs based on the class of cell wall glycan they each recognize.

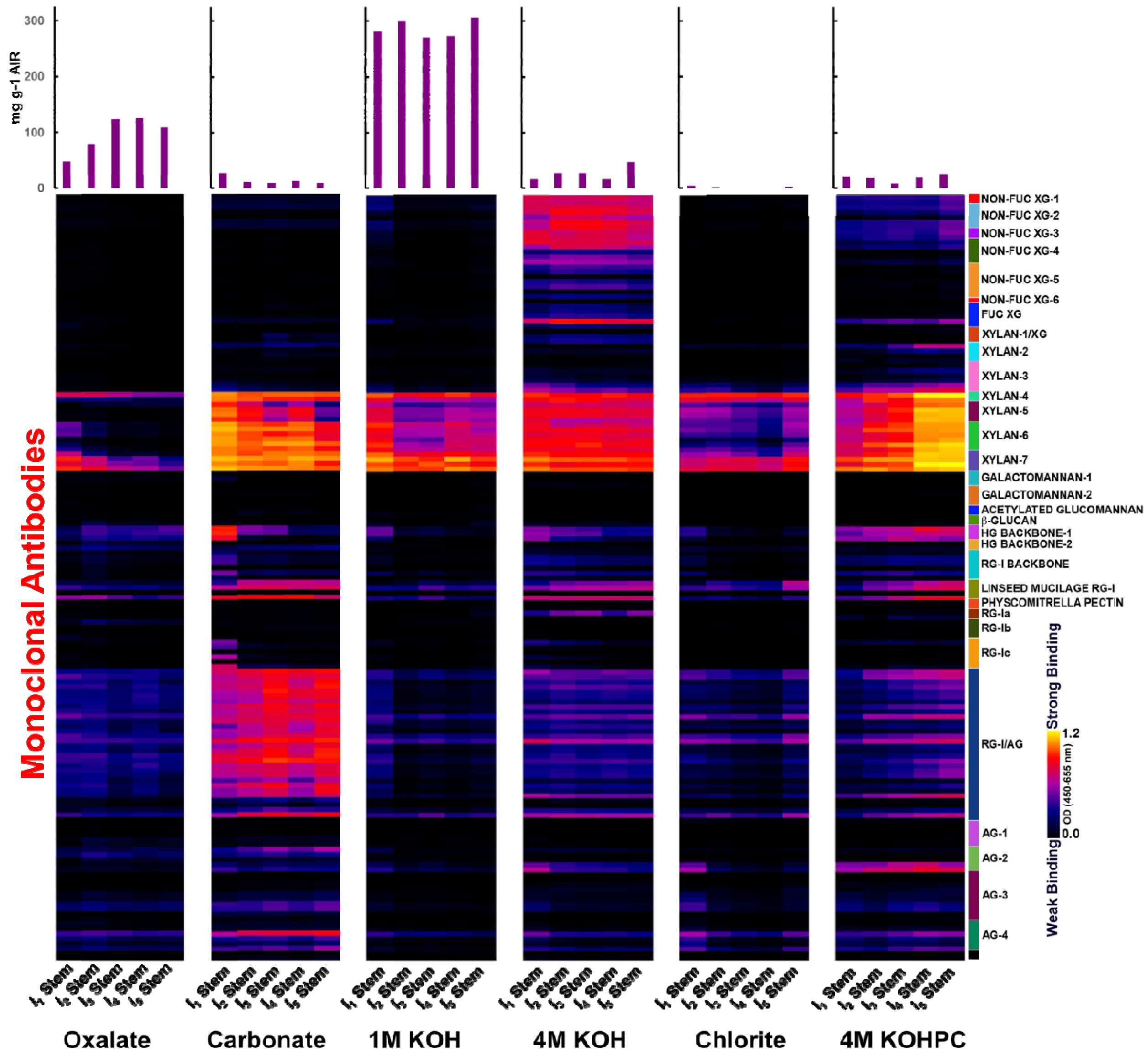

Supplemental Figure S3. Heat map abundance of major non-cellulosic glycan epitopes in oxalate, carbonate, 1M KOH, 4M KOH, chlorite, and 4M KOHPC extracts from stem internodes I_1-I_5. Extracts were screened by ELISA using a comprehensive suite of cell wall glycan-directed mAbs as described in materials and methods. Binding response values are depicted as heat maps with black-red-bright yellow color scheme representing from no binding to strongest binding. The amount of carbohydrate material recovered per gram of AIR is depicted in the bar graphs (purple) above the heat maps. The panel on the right-hand side of the heat map shows the groups of mAbs based on the class of cell wall glycan they each recognize.
